# Evidence of new species for malaria vector *Anopheles nuneztovari* sensu lato in the Brazilian Amazon region

**DOI:** 10.1186/s12936-016-1217-6

**Published:** 2016-04-12

**Authors:** Vera Margarete Scarpassa, Antonio Saulo Cunha-Machado, José Ferreira Saraiva

**Affiliations:** Laboratório de Genética de Populações e Evolução de Mosquitos Vetores de Malária e Dengue, Coordenação de Biodiversidade, Instituto Nacional de Pesquisas da Amazônia (INPA), Avenida André Araujo, 2936. Bairro Petrópolis, Manaus, Amazonas 69067-375 Brazil; Programa de Pós–Graduação em Genética, Conservação e Biologia Evolutiva, Instituto Nacional de Pesquisas da Amazônia (INPA), Avenida André Araujo, 2936. Bairro Petrópolis, Manaus, Amazonas 69067-375 Brazil

**Keywords:** Cryptic species complex, Evolutionary genetics, Molecular entomology, Malaria vector

## Abstract

**Background:**

*Anopheles nuneztovari* sensu lato comprises cryptic species in northern South America, and the Brazilian populations encompass distinct genetic lineages within the Brazilian Amazon region. This study investigated, based on two molecular markers, whether these lineages might actually deserve species status.

**Methods:**

Specimens were collected in five localities of the Brazilian Amazon, including Manaus, Careiro Castanho and Autazes, in the State of Amazonas; Tucuruí, in the State of Pará; and Abacate da Pedreira, in the State of Amapá, and analysed for the *COI* gene (Barcode region) and 12 microsatellite loci. Phylogenetic analyses were performed using the maximum likelihood (ML) approach. Intra and inter samples genetic diversity were estimated using population genetics analyses, and the genetic groups were identified by means of the ML, Bayesian and factorial correspondence analyses and the Bayesian analysis of population structure.

**Results:**

The Barcode region dataset (N = 103) generated 27 haplotypes. The haplotype network suggested three lineages. The ML tree retrieved five monophyletic groups. Group I clustered all specimens from Manaus and Careiro Castanho, the majority of Autazes and a few from Abacate da Pedreira. Group II clustered most of the specimens from Abacate da Pedreira and a few from Autazes and Tucuruí. Group III clustered only specimens from Tucuruí (lineage III), strongly supported (97 %). Groups IV and V clustered specimens of *A. nuneztovaris.s.* and *A. dunhami*, strongly (98 %) and weakly (70 %) supported, respectively. In the second phylogenetic analysis, the sequences from GenBank, identified as *A. goeldii,* clustered to groups I and II, but not to group III. Genetic distances (Kimura-2 parameters) among the groups ranged from 1.60 % (between I and II) to 2.32 % (between I and III). Microsatellite data revealed very high intra-population genetic variability. Genetic distances showed the highest and significant values (*P* = 0.005) between Tucuruí and all the other samples, and between Abacate da Pedreira and all the other samples. Genetic distances, Bayesian (Structure and BAPS) analyses and FCA suggested three distinct biological groups, supporting the barcode region results.

**Conclusions:**

The two markers revealed three genetic lineages for *A. nuneztovaris.l.* in the Brazilian Amazon region. Lineages I and II may represent genetically distinct groups or species within *A. goeldii*. Lineage III may represent a new species, distinct from the *A. goeldii* group, and may be the most ancestral in the Brazilian Amazon. They may have differences in *Plasmodium* susceptibility and should therefore be investigated further.

**Electronic supplementary material:**

The online version of this article (doi:10.1186/s12936-016-1217-6) contains supplementary material, which is available to authorized users.

## Background

*Anopheles nuneztovari* sensu lato was originally described in San Carlos, State of Cojedes, western Venezuela. It is geographically distributed from eastern Panama to northern South America [1] and is considered one of the four most important malaria vectors in northern South America, together with *A. darlingi*, *A. albimanus* and *A. aquasalis* [2, 3]. *Anopheles nuneztovaris.l.* has been long recognized to be an important human malaria vector in Colombia and Venezuela, presenting endo and exophagic behaviours, besides high levels of anthropophily and infection rate [4, 5]. Differently from Colombia and Venezuela, the Brazilian populations of this species, which are predominantly zoophagic, were not considered a malaria vector in past decades (1940s–1970s). However, with the development of more sensitive techniques for detecting malaria parasites, *A. nuneztovaris.l.* has been reported to be infected with *Plasmodium* species in five states of the Brazilian Amazon region [6–12], and was recently considered an important local vector in the State of Amapá, Brazil [12]. Supporting these findings, experimental infection studies conducted with *A. nuneztovari s.l.* from Manaus (MN), Brazil, reported a high infection rate for *Plasmodium vivax* [13]. In fact, the “populations” of the Brazilian Amazon region feed preferentially on bovines rather than humans, and this behaviour is probably the limiting factor to transmitting human malaria [12].

In view of its importance as malaria vector in northern South America, a great number of studies was conducted with *A. nuneztovaris.l.* from Colombia/Venezuela (malaria vector) and the Amazon Basin (non-malaria vector) [14–24], aiming to understand the distinct patterns of malaria transmission across its geographic range. The results indicated that *A. nuneztovaris.l.* could encompass two ecologically and genetically distinctive geographic populations, but no strong evidence of one cryptic species complex—as previously thought—was found, reflecting a very recent evolutionary history [14–24].

Based on phylogenetic analyses of three molecular markers and the comparison of the male genitalia aedeagus apex, Calado et al. [25] demonstrated that the *A. nuneztovari* from Colombia and Venezuela are likely to be distinct from the Brazilian Amazon specimens studied by the authors [25]. Considering these results, *A. goeldii* was revalidated from synonymy with *A. nuneztovari* [25]. Scarpassa [26] observed differences in the length of the pre-humeral dark spot (PHD) and the length of the subcostal pale spot (SCP) on the wings of the adult females from Buenaventura and Tibú (Colombia) compared with the specimens of the Brazilian Amazon, and—based on a taxonomic key—they were identified as *A. rangeli*. However, the 4th instar larvae, male genitalia and eggs were identified as *A. nuneztovari*. Sant’Ana et al. [27] reported similar findings in the revision of *A. goeldii*. Recently, *A. dunhami* was also included in *A. nuneztovaris.l*., based on phylogenetic analyses performed with three markers [28]. Thus, the taxonomic status of *A. nuneztovaris.l.* now includes: *A. nuneztovaris.s.* which occurs in Colombia and western Venezuela, *A. goeldii* in the Brazilian Amazon region, and *A. dunhami,* found in the Brazilian Amazon [25, 29–31] and Colombia [32]. In the Brazilian Amazon, however, *A. dunhami* shows overlap with *A. goeldii* in a large geographic area [31]. The role of *A. nuneztovaris.s.* as malaria vector has been elucidated, but the role of each lineage and/or *A. goeldii* species within the Brazilian Amazon still remains to be clarified, despite the reports of infection by *Plasmodium* spp. [6–12]. *Anopheles dunhami* is abundant in forests and has zoophilic behaviour [33], and it was never found infected with the malaria parasite, or was wrongly identified as *A. goeldii* or other *A. nuneztovari s.l*. lineages.

Along with the presence of three cryptic species in *A.nuneztovaris.l*., two genetic lineages were reported in the Brazilian Amazon, based on ITS2 sequences [17] and mtDNA-RFLP [18, 23]. More recently, Mirabello and Conn [24] detected five lineages with the *white* gene, three of which (1, 4 and 5) in the Brazilian Amazon + Suriname, and two (2 and 3) in Colombia/Venezuela. The two lineages observed in Colombia and Venezuela could correspond to *A. nuneztovaris.s*., whereas the three of the Brazilian Amazon + Suriname could correspond to *A. goeldii* [24]. Scarpassa and Conn [31] proposed the existence of four lineages across its geographic range, based on the fragment at the 3′ end of the *COI* gene. Specimens from Bolivia/Colombia/Venezuela grouped to a single cluster (subclade II-C), and may represent *A. nuneztovari s.s*. The specimens from the Brazilian Amazon + Suriname grouped to three clusters (Clade I and subclades II-A and II-B) and may represent *A. goeldii* and other species within *A. nuneztovari s.l*. The inconsistence in the number of lineages among studies [17, 18, 23, 24, 31] could be related especially to sampling strategies within the Brazilian Amazon region.

Given the evidence above, there is no doubt that *A. nuneztovari* consists of a cryptic species complex in northern South America, and that the Brazilian populations comprise two or more genetic lineages or species. Therefore, analyses with multi-markers will be needed to test the hypothesis of multiple species in the Brazilian Amazon region. The definition of these lineages could help understanding how they contribute to the malaria transmission in this region, especially because they may show differences in ecology, behavior and *Plasmodium* susceptibility, with consequent implications on management, surveillance and control measures. The aim of this study was to investigate whether the *A. nuneztovaris.l*. lineages detected [17, 18, 23, 24, 31] should actually have species status, based on the analyses of specimens from five localities of the Brazilian Amazon with the mitochondrial (barcode region) and nuclear (12 microsatellites loci) markers. Two of the five localities [Autazes (AU), Abacate da Pedreira (AP)] were sampled for the first time in this study, whereas the other localities [MN, Careiro Castanho (CS), Tucuruí (TU)] were previously studied by Scarpassa and Conn [31], using a fragment of the 3′ end of the *COI* gene. This fragment has demonstrated to be highly variable in anopheline species, being therefore amply used in the population genetics and phylogeographic studies of this group [34]. In contrast, the barcode region (Folmer region) that consists of a 648 bp fragment at the 5′ end of the *COI* gene has emerged as the standard barcode region, because it presents a low rate of intra-specific and high inter-specific variation (barcoding gap), thus permitting the characterization of each taxonomic group or unit [35]. This region has shown to be a valuable tool in the identification of species complexes in anophelines [36–38]. The microsatellite markers, developed and characterized previously for *A. nuneztovaris.l*. [39, 40], were analysed for the first time in the present study. These markers are appropriate to estimate intra-population genetic diversity, fine-scale population structure, and also for taxonomic and evolutionary genetic studies of very recently evolved species. In the present study, the samples analysed were named *A. nuneztovaris.l*.

## Methods

### Sample collection

**Fig. 1:**
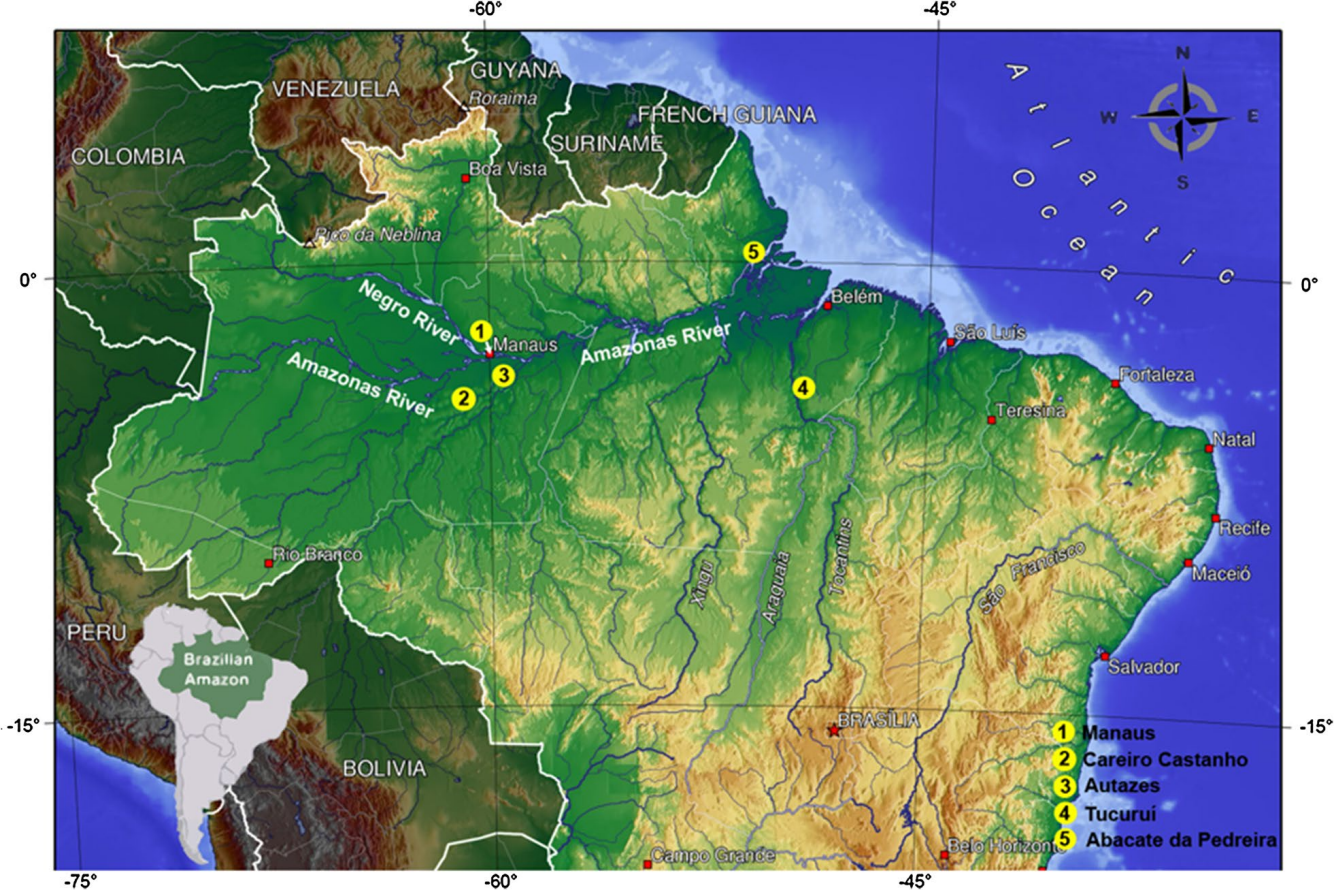
Collection sites of the five *Anopheles nuneztovari* *s.l.* samples in the Brazilian Amazon region

*Anopheles nuneztovari s.l.* specimens were collected in five localities of the Brazilian Amazon region (Table 1; Fig. 1), as follow: MN, CS and AU in the State of Amazonas; TU in the State of Pará; and AP in the State of Amapá, Brazil. All information regarding collection data, State, coordinates and sample size for each marker is shown in Table 1. The localities of MN, CS and AU are situated in the central region of the Brazilian Amazon, TU in the east and AP in the northeast. The collections were authorized by the Brazilian Institute for the Environment and Renewable Natural Resources (IBAMA) and by the System of Authorization and Information in Biodiversity (SISBIO), license number 38440-1 awarded to VMS.

**Table 1:** Information on the *Anopheles nuneztovari* *s.l.* collection sites in the Brazilian Amazon region and Sitronela, in Colombia

Localities, state	Abbreviation	Coordinates (Lat., long.)	Sample size *COI*	Sample size MSTL	Collection date
Manaus, Amazonas	MN	03° 03′ S; 59° 51′ W	12	32	June, 2010
Careiro Castanho, Amazonas	CS	03° 49′ S, 60° 21′ W	22	32	July, 2010
Autazes, Amazonas	AU	03º 41′ S; 59º 07′ W	27	32	May, 2013
Tucuruí, Pará	TU	03° 42′ S, 49° 27′ W	14	32	August and October, 1992
Abacate da Pedreira, Amapá	AP	00° 07′ S; 51° 17′ W	28	32	February, 2013
Subtotal			103	160	
*Anopheles nuneztovari* s.s.^a^					
Sitronela, Valle^b^	SI	03º49′ N, 77º 04′ W	8	NA	June, 1994
*Anopheles dunhami* ^a^					
Autazes, Amazonas	AU	03º 41′ S; 59º 07′ W	2	NA	May, 2013
Coari, Amazonas	CO	04° 05′ S, 63° 07′ W	1	NA	June, 2002
Manaus, Amazonas	MN	03° 03′ S; 59° 51′ W	1	NA	June, 2013
Total			115	160	

*COI* Cytochrome oxidase, *I* subunit, *MSTL* microsatellites, *NA* not analysed

^a^ Species included in the phylogenetics analyses and genetic distances analysis

^b^ The Sitronela locality is situated in Buenaventura, Department del Valle, in Colombia

Adult female mosquitoes were collected with Shannon traps and/or while resting on cattle corrals, transferred into labelled cups and transported to the laboratory. At the laboratory, the mosquitoes (females) were individually isolated in plastic cups for egg laying and the offspring were reared until they became adults. The morphological identification was done on adults (females) and eggs, as described in Faran [1] and Cova-Garcia [41]. Specimens were preserved in ethanol 95 % and stored at −20 °C or dried and stored at −80 °C until DNA extraction.

### DNA extraction, PCR and sequencing

Genomic DNA was extracted individually from whole mosquitoes, using the phenol and chloroform method [42], resuspended in 30 μL of 1 × TE buffer (10 mM Tris–Cl pH 8.0, 1 mM EDTA pH 8.0) or sterile water, and then stored at −80 °C until amplification by polymerase chain reaction (PCR) for the two markers.

For the mitochondrial DNA analyses, a 663 bp (base pair) fragment encompassing the barcode DNA region (Folmer region) of the *COI* gene of 103 individuals was amplified, as shown in Table 1. The primers (10 μM) used were LCO 1490 and HCO 2198 [43], and the amplification conditions were as described in Folmer et al. [43]. All PCR reactions included negative controls. The PCR products were visualized on 1 % agarose gels under UV light and purified with PEG 8000. Both DNA strands of the purified PCR product were electro-injected into an automated ABI 3130 xl Genetic Analyzer sequencer (Applied Biosystems, Thermo Fisher Scientific, Waltham, MA, USA), available at INPA (MN, Brazil).

### Microsatellite analyses

In the microsatellite analyses, 32 specimens per locality were genotyped for the 12 loci under study (Table 1). Of these 12 loci, nine are described in Austin et al. [39]: *Anu1, Anu4*, *Anu6*, *Anu9*, *Anu10*, *Anu12*, *Anu14*, *Anu15* and *Anu16*; the other three are described in Cunha-Machado and Scarpassa [40]: *Anu22*, *Anu25* and *Anu28*.

The microsatellite loci were amplified by PCR in a final volume of 10 µL reaction solution containing 10–20 ng DNA template, 1.0 μL 10× buffer, 2.1 μL 1 mM dNTPs, 0.3 µL 50 mM MgCl_2_, 0.4 μL 4 mM M13-tailed forward primer [44], 0.4 μL 4 mM M13-labelled primer (FAM, HEX and TAMRA), 0.8 μL 4 mM reverse primer, 0.2 μL 5 U/µL Platinum Taq DNA Polymerase (Invitrogen Inc., Carlsbad, CA, USA), and 3.8 μL sterile water to complete the total volume. PCR was carried out in 2 steps: the first step consisted of denaturation (68 °C for 2 min, 95 °C for 30 s) followed by 30 cycles of 35 s at 92 °C, 35 s at the primer-specific annealing temperature [39, 40], and 35 s at 72 °C.

The second step consisted of 15 cycles of 30 s at 92 °C, 35 s at 53 °C, 35 s at 72 °C, followed by a final extension at 72 °C for 30 min. The PCR fragments were analysed in an automated ABI 3130 xl Genetic Analyzer (Applied Biosystems, Thermo Fisher Scientific, Waltham, MA, USA), and the allele sizes were scored using GeneScan 500 ROX dye (Applied Biosystems) and genotyped in the GeneMapper version 4.0 (Applied Biosystems) program.

### Statistical analyses

#### Barcode region analyses

The forward and reverse sequences were automatically aligned in the Clustal W program and manually edited in Bioedit v. 7.0.8.0 [45], and the chromatograms were corrected using the electropherogram viewer Chromas Lite^®^ [46]. The consensus sequences generated had a large (~625 bp) region of overlap. The consensus sequences of each specimen were confirmed using BLAST (Basic Local Alignment Search Tool) [47]. The haplotypes were determined using the DnaSP v. 5.10 [48] and the TCS v. 1.21 [49] programs. Identical sequences were considered to be a single haplotype. To analyse population history patterns, a haplotype network was generated by means of a parsimony-based method that calculates the maximum number of mutational connections between pairs of sequences by the 95 % parsimony criterion using the TCS program, v. 1.21 [49].

Phylogenetic relationships among the 27 haplotypes were inferred using the maximum likelihood (ML) method in the Mega program, v. 6 [50], with 1000 replicates. This analysis was performed using the general time reversible (GTR) + G + I nucleotide substitution model, previously selected with the Akaike Information Criterion (AIC) in the jModelTest [51]. For this analysis, eight specimens of *A. nuneztovaris.s.* from Sitronela, Buenaventura, Colombia, and four specimens of *A. dunhami* from three localities of the Brazilian Amazon region (Table 1) were also sequenced. These sequences (haplotypes) together with the 27 haplotypes of this study were included as ingroup in the analysis. Sequences of *A. oswaldoi* species A were used as outgroup.

A second phylogenetic analysis was carried out using the 27 haplotypes of this study, two haplotypes of *A. nuneztovaris.s*., three haplotypes of *A. dunhami* and the sequences from other localities of the Brazilian Amazon region (EU848313–EU848332) published by Calado et al. [25] and Colombia (AF368078, AF368089, AF368094, AF368106, AF368115) available in GenBank. Our sequences had a length of 663 bp, whereas those of Calado et al. [25] were 493 bp long. To build the input file with two datasets, a fragment of ~196 bp from our sequences was removed, as well as a ~15 bp end fragment of the sequences from Calado et al. [25] and from GenBank. The final input file comprised sequences of 467 bp in size, and therefore some informative sites were lost. This analysis was inferred using ML in Mega v. 6 [50] with the same parameters cited above. Sequences of *A. oswaldoi* species A were used as outgroup. A Bayesian inference (BI) analysis was attempted, but the tree topology was not well resolved; therefore, the results were not included in this study.

The intra-population and overall genetic diversity measurements, such as haplotype numbers (*NH*), transition and transversion rates (*T*s/*T*v), number of segregating sites (*NS*), number of private sites (*NPS*), average number of nucleotide differences (*K*), haplotype (*h*) and nucleotide (*π*) diversities, were estimated using the DnaSP v. 5.10 [48] and Arlequin v 3.1 [52] programs. Neutrality tests, Tajima’s *D* [53], Fu and Li’s *D* and *F* [54] and Fu’s *F*s [55] were inferred in DnaSP v. 5.10 [48] and Arlequin, v 3.1 [52]. Tajima’s *D* [53], Fu and Li’s *D* and *F* tests [54] were used to test the hypothesis that all mutations are selectively neutral. Tajima’s *D* test is based on the differences between the number of segregating sites and the average number of nucleotide differences. The *D* and *F* tests, proposed by Fu and Li, are based on molecular polymorphism data. Fu’s *F*_S_ test [55] assesses the haplotype structure based on the haplotype frequency distribution and was used as an additional neutrality test. This test is more powerful for detecting population expansion and genetic hitchhiking, whereas Tajima’s *D*, Fu and Li’s *F* and *D* tests are the most effective ones for detecting background selection.

Population genetic structure was estimated using traditional genetic differentiation measurements (*Φ*_ST_ pairwise) and hierarchical analysis of molecular variance (AMOVA). Both estimates were made in Arlequin, v.3.1 [52], and the significance level was inferred by permutation tests (10,000 replicates). AMOVA was performed on two different levels of population structure, to partition total molecular variance: (1) all samples (non-grouped) to test the overall differences among samples; and (2) to test the subdivision level between the central (MN, CS, AU) and eastern/northeastern (TU, AP) Brazilian Amazon regions.

The intra and inter-samples genetic distances of the five localities and among the three lineages were calculated using Mega v.6.0 [50], based on the Kimura-2 parameters (K-2P) evolutionary model. For these calculations, the lineages generated in Fig. 3 were used. The divergence time among the lineages was also calculated using the divergence sequence (*D*_xy_) and the mutation rate of 2.3 % per million years [56], often estimated for mtDNA in insects. The haplotypes sequences of the five *A. nuneztovaris.l.* collection localities as well as those of *A. nuneztovaris.s.* and *A. dunhami* generated in this study are deposited in GenBank under accession numbers: KU865529 to KU865555 (H1 to H27), KU865556 to KU865557 (H28, H29) and KU865558 to KU865561 (H30 to H33).

#### Microsatellite analyses

The dataset was checked in the Micro-Checker v.2.2.3 [57] to detect potential errors that might have occurred at each locus during the genotyping, such as stuttering, large allele dropouts and null alleles. Whenever null alleles occurred, their frequencies were calculated in the same program. The intra-population genetic diversity measurements, such as the observed (*H*_O_) and expected (*H*_E_) heterozygosities, the Hardy–Weinberg equilibrium (HWE) and linkage disequilibrium (*LD*) were estimated using Arlequin, v.3.1 [52]. The number of alleles per locus (*N*_A_) and number of private alleles were calculated in Genalex, v.6.41 [58], whereas the inbreeding coefficient (*F*_IS_) and allele richness (*A*_R_) were estimated in Fstat, v.2.9.3 [59].

The genetic structure was accessed using pairwise *F*_ST_ and AMOVA. Both analyses were estimated in Arlequin, v.3.1 [52], with significance levels of 10,000 permutations. In the AMOVA test, the same hierarchical levels estimated for the Barcode region were used for the microsatellite data, as described above.

The STRUCTURE program, v. 2.3 [60], was used through a Bayesian approach to test the population structure among the samples. This method distinguishes clusters of genetically similar individuals from multilocus genotypes, without prior knowledge of their population affinities and origin, assuming an admixture model that allows individuals to have ancestors from more than one biological group. The model assumes *K* genetic clusters, each one having a characteristic set of allele frequencies at each locus. Thus, an admixture model with correlated allele frequencies was assumed. The analysis was performed for genetic clusters (*K*) ranging in number from 1 to 6. Consistent results across runs were obtained using a burn-in period of 100,000 permutations, followed by 1000.000 Markov Chain Monte Carlo (MCMC) repeats. The true number of populations is expected to be the value of K that maximizes the estimated model log-likelihood, log [P(X|K)] [61]. For the most likely value of K, the proportion of allocation (*Q*) of the localities sampled within detected groups and the individual allocation ratio *q* (proportion of ancestral genome of each specimen in the group) were estimated. Furthermore, the genetic structure was accessed by factorial correspondence analysis (FCA) that used the individual multilocus scores, computed in the Genetix program [62]. The genetic structure was again accessed by Bayesian analysis of population structure (BAPS) [63]. In this analysis, 1–5 clusters were employed (the upper corresponding to the total number of sampled localities), and five independent runs were implemented. The most probable genetic cluster configuration was prepared by comparing the log-likelihood values of the best models.

The Bonferroni correction [64] was applied to *P* values in all the statistical analyses of this study that involved multiple comparisons.

## Results

### Barcode region analysis

The dataset consisted of 103 sequences with a fragment size of 663 bp (Table 1). The amino acid translations revealed no stop codons, ensuring the absence of nonfunctional genes (pseudogenes) in the dataset. All sequences had 39 variable sites, 35 of which were parsimoniously informative. Of the 94 nucleotide substitutions, 87 (92.55 %) were transitions and seven (7.45 %) transversions. The nucleotide composition was rich in A + T (Mean = 67.70 %), especially in the third codon position (92 %). In this study, there was evidence of heteroplasmy in two of the 16 individuals (12 %) from TU. Double picks (adenine/guanine) were observed at position 435 of the consensus sequences, and in both forward and reverse sequences. These sequences were excluded from the statistical analyses; therefore, the sample size from TU consisted of n = 14 (Table 1).

**Fig. 2:**
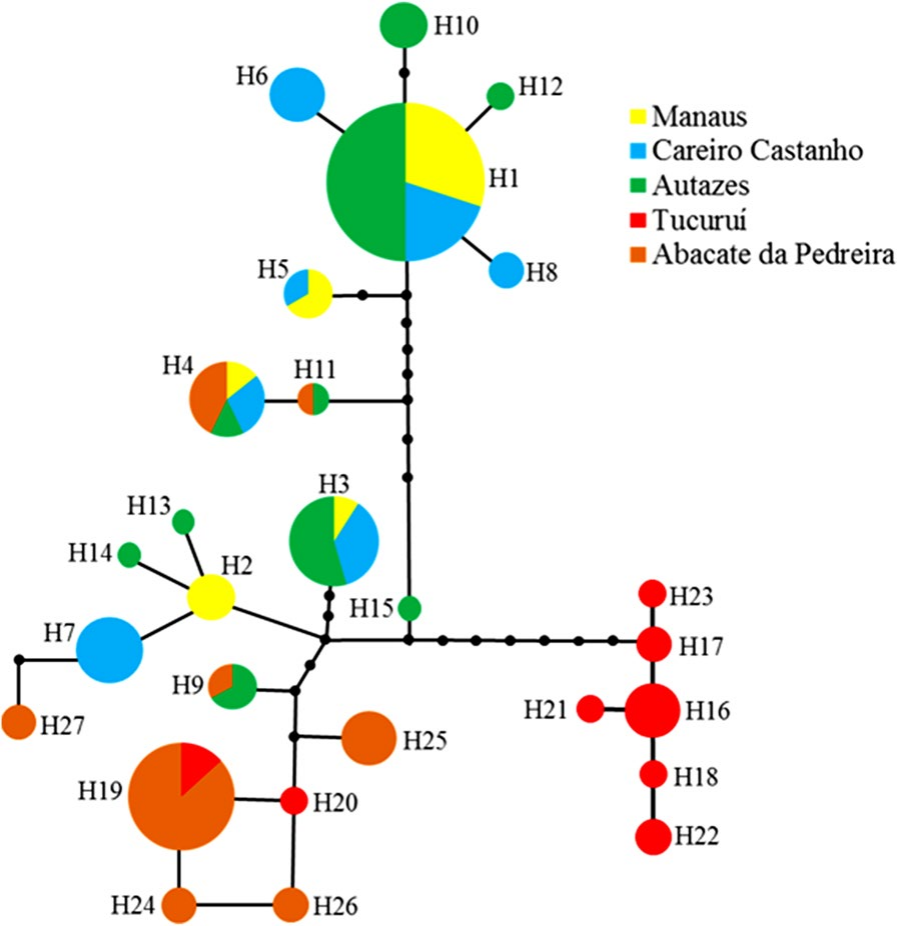
Parsimony haplotype network of the 27 *Anopheles nuneztovari* s*.l.* haplotypes from the Brazilian Amazon region. H1–H27, haplotypes; H1, likely the ancestral haplotype. The haplotype circle sizes are proportional to the number of individuals observed for each haplotype (see Table 2). *Filled smaller circles* represent mutational events

Of the 27 haplotypes observed, seven (25.93 %) were shared among samples and 20 (74.07 %) were singletons and/or exclusive of each sample (Table 2). Autazes had the largest number of haplotypes (10) and MN the lowest (5). Haplotypes H1 (likely the ancestral), H3 and H19 were the most common. H1 and H3 were shared among the three samples from the State of Amazonas. H19 was shared by 13 specimens from AP and two from TU. The sample from TU did not share haplotypes with three samples from the State of Amazonas (Table 2; Fig. 2).

**Table 2:** Haplotype frequency observed for the *COI* gene (Barcode region) in the five *Anopheles nuneztovari* *s.l.* samples from the Brazilian Amazon region

Samples	*N*	Haplotype frequency
Manaus	12	*H1(6)*, H2(2), *H3(1)*, *H4(1)*, *H5(2)*
Careiro Castanho	22	*H1(4)*, *H3(4)*, *H4(2)*, *H5(1)*, H6(4), H7(5), H8(2)
Autazes	27	*H1(10)*, *H3(6)*, *H4(1)*, *H9(2)*, H10(3), *H11(1)*, H12(1), H13(1), H14(1), H15(1)
Tucuruí	14	H16(4), H17(2), H18(1), *H19(2)*, H20(1), H21(2), H22(1), H23(1)
Abacate da Pedreira	28	*H4(3)*, *H9(1)*, *H11(1)*, *H19(13)*, H24(2), H25(4), H26(2), H27(2)
Total	103	

In parentheses, number of individuals observed for each haplotype. The italics haplotypes are shared among samples

*N* number of specimens sequenced

The haplotype network (Fig. 2) suggested three lineages. Lineage I comprised most haplotypes from MN, CS and AU and few haplotypes from AP (H4 = 3 specimens; H11 = 1 specimen). Lineage II clustered haplotypes from all samples, including two of the most common haplotypes (H3 and H19). Lineage III consisted only of haplotypes from TU (H16–H18, H21–H23 = 11 specimens), which was separated from lineages I and II by 11–16 mutational steps and by 9–12 mutational steps, respectively.

**Fig. 3:**
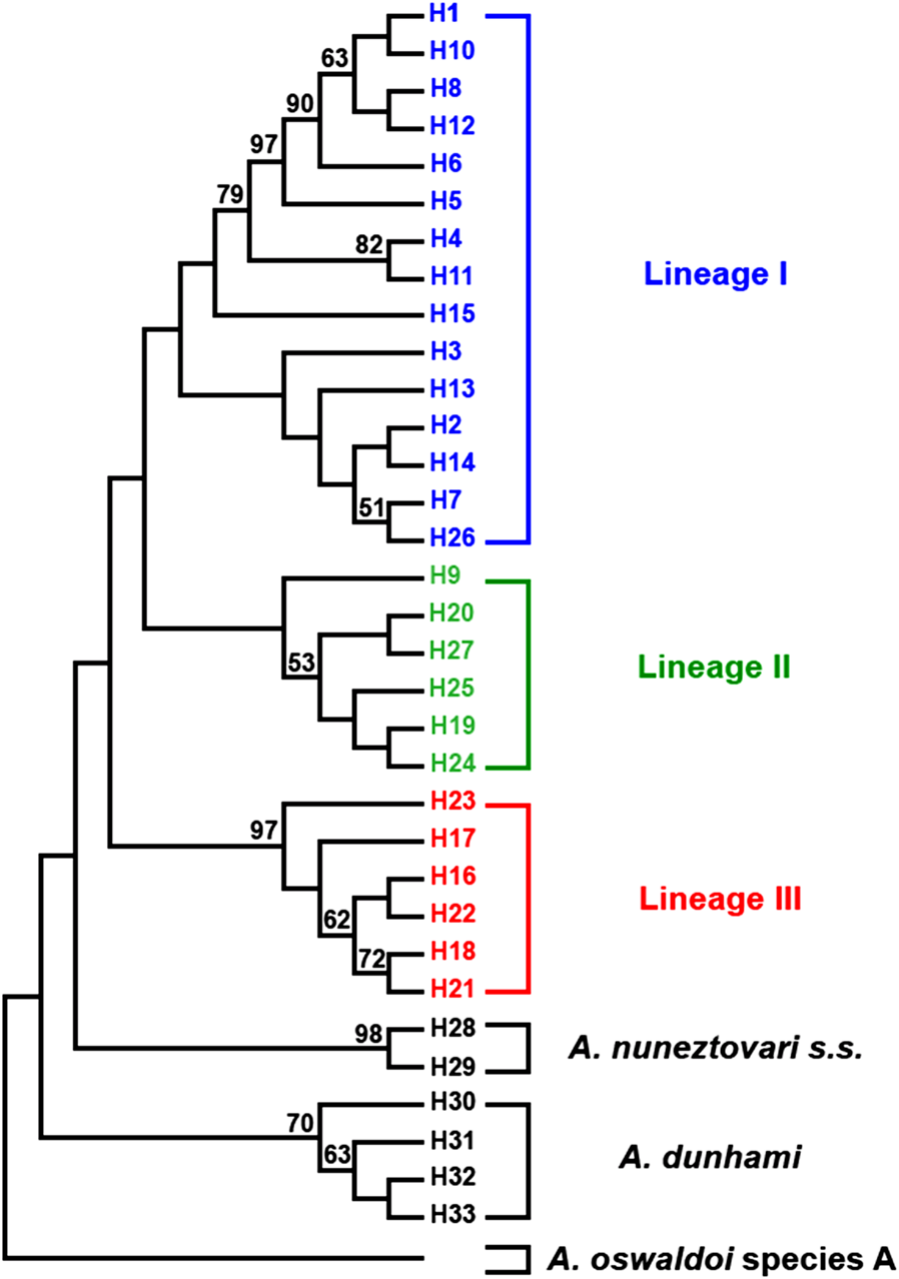
Maximum Likelihood (ML) topology tree of the 27 *Anopheles nuneztovari* *s.l.* haplotypes from the Brazilian Amazon region, two *Anopheles nuneztovari* *s.s.* haplotypes (H28, H29, both from Sitronela, Colombia; see Table 1) and four *Anopheles dunhami* haplotypes (H30 from AU, H31 from Coari, H32 from MN, H33 from AU; see Table 1), using the GTR + I + G nucleotide substitution model. Values above each branch represent bootstrap support. *Anopheles oswaldoi* species A was used as outgroup

The ML tree retrieved five groups (Fig. 3) suggesting monophyly. Groups I and II were weakly supported. Group I clustered all specimens from MN and CS, most of those from AU and six specimens (H4, H11, H26) from AP. Group II clustered most of the specimens from AP, two specimens (H9) from AU and three (H19, H20) from TU. Group III clustered only specimens from TU (lineage III) and was strongly supported (97 %). Haplotypes of *A. nuneztovari* s.s. and *A. dunhami* clustered in separated branches, with 98 and 70 % bootstrap support, respectively. Groups I and II generated in this analysis did not correspond to lineages I and II visualized in the haplotype network (Fig. 2); this discrepancy may be explained by little resolution of these groups in the ML tree (weakly supported), likely due to the small number of informative sites between them for this marker.

In the second phylogenetic analysis (Additional file 1), in which the sequences downloaded from GenBank were included, the ML tree retrieved six monophyletic groups. All sequences identified as *A. goeldii* from GenBank clustered with the haplotypes of groups I and II of this study (Fig. 3), forming three distinct groups (I, III, V), but all weakly supported. In the internal group, *A. dunhami* (group VI) constituted a sister group of the monophyletic clade formed by *A. goeldii* (Groups I, III, V), *A. nuneztovaris.s.* (Group II) and group IV (lineage III of this study). Of the three *A. goeldii* groups, group V is likely the most basal one and may actually be a distinct species. This group is a sister group to a monophyletic clade formed by groups I and III (*A. goeldii*), and II (*A. nuneztovaris.s*.) and IV (lineage III). This topology suggests that *A. goeldii* does not have an exclusive ancestry and could be paraphyletic. Groups I and III (*A. goeldii*) and II (*A. nuneztovaris.s*.) formed a monophyletic clade that is a sister group of group IV. The latter was well supported (88 %) and may also represent a distinct species. In this context, *A. goeldii* (groups I and III) could be a synonym of *A. nuneztovaris.s.* (group II), or *A. goeldii* (group I) could be a synonym of *A. nuneztovaris.s.* and *A. goeldii* (group III), a distinct species, or even each group could be a distinct species. Both trees were rooted using *A. oswaldoi* species A as outgroup.

Table 3 shows the intra-sample genetic diversity measurements. The haplotype diversity ranged from 0.742 ± 0.116 (MN) to 0.901 ± 0.058 (TU). The nucleotide diversity ranged from 0.00601 ± 0.00129 (AP) to 0.01207 ± 0.00082 (CS). Tucuruí had the highest number of private sites (9); in the other samples it varied from 0 (MN) to 4 (AU). Table 3 also shows the neutrality tests for the five samples and total data. None of these tests produced statistically significant results, except for the sample from CS, for which Fu’s *F*s test was positive and significant (4.082; *P* < 0.05), indicating balancing selection, recent bottleneck or cryptic population structure.

**Table 3:** Intra-population genetic diversity and neutrality tests estimated for the *COI* gene in the five *Anopheles nuneztovari* s.l. samples from the Brazilian Amazon region

Samples	*T*s/*T*v	*NH*	*S*	*NPS*	*K*	*H* ± SD	*π* ± SD	Tajima’s *D*	Fu and Li’s *D*	Fu and Li’s *F*	Fu’s *F*s
Manaus	17/2	5	19	0	6.44	0.742 ± 0.116	0.00971 ± 0.00207	0.1028	0.1943	0.1939	3.020
Careiro Castanho	20/2	7	22	2	8.00	0.870 ± 0.033	0.01207 ± 0.00082	1.2233	1.1029	1.3288	4.082*
Autazes	20/2	10	22	4	7.66	0.818 ± 0.056	0.01155 ± 0.00089	1.2271	1.0867	1.3266	1.767
Tucuruí	15/0	8	15	9	5.20	0.901 ± 0.058	0.00784 ± 0.00198	0.4159	0.9133	0.8925	−0.321
Abacate da Pedreira	15/1	8	16	1	3.99	0.762 ± 0.073	0.00601 ± 0.00129	−0.1046	1.1925	0.9242	0.907
Total	17.4/1.4	27	39	3.20	9.28	0.9210 ±0.014	0.01399 ±0.00042	0.7419	0.9768	1.0586	−1.260

*T*s/*T*v transitions/transversions, *NH* Number of haplotypes, *S* Number of segregating sites, *NPS* Number of private sites, *K* Average number of nucleotide differences, *H* ± SD and *π* ± SD Haplotype and nucleotide diversities, respectively, with respective standard deviations (SD)

* *P* < 0.05

Low genetic differentiation (*Φ*_ST_ = −0.01162 to −0.01975) and extensive gene flow (*N*m = infinity) were observed among the three samples from the State of Amazonas, indicating genetic homogeneity (data not shown). In contrast, great and significant genetic differentiation was found between MN/CS/AU and TU (*Φ*_ST_ = 0.5988; 0.5146; 0.5231, respectively; *P* = 0.00000 ± 0.0000) and between MN/CS/AU/TU and AP (*Φ*_ST_ = 0.5376; 0.4141; 0.4116; 0.5459, respectively; *P* = 0.00000 ± 0.0000). In all comparisons, the *N*m values were <1, suggesting absence of gene flow. AMOVA analysis including all samples also revealed significant structure between groups (*Φ*_ST_ = 0.3800; *P* = 0.00000 ± 0.0000). High genetic structure was found between two groups (*Φ*_CT_ = 0.3093), but it was not statistically significant (*P* = 0.09677 ± 0.01008) (Table 4).

**Table 4:** Analysis of molecular variance (AMOVA) for testing the hierarchical population structure within *Anopheles nuneztovari s.l.* from the Brazilian Amazon region

*COI*					Microsatellites			
Groups tested	Source of variation components	d.f.	Percentage variance (%)	Fixation index	Source of variation components	d.f.	Percentage variance (%)	Fixation index
*No grouping (all)* MN, CS, AU, TU, AP	Among samples	4	38.00	*Φ*_*ST*_ = 0.3800***	Among samples	1	8.08	*F*_*ST*_ = 0.081***
	Within samples	98	62.00		Within samples	315	91.92	
*Two groups* (1) MN, CS, AU (2) TU, AP	Between groups	1	30.93	*Φ*_*CT*_ = *0.3093*	Between groups	1	6.03	*F*_CT_ = 0.060
	Among samples within groups	3	14.37	*Φ*_*SC*_ = 0.2081***	Among samples within groups	3	4.27	*F*_*SC*_ = 0.043***
	Within samples	98	54.70	*Φ*_*ST*_ = 0.4530***	Within samples	315	89.70	*F*_*ST*_ = 0.103***

See Table 1 for locality abbreviations

*Significance test* 10,000 permutations, *d.f.* degrees of freedom, *Φ*_*ST*_ fixation index within samples, *Φ*_*CT*_ fixation index between regions, *Φ*_*SC*_ fixation index among samples within regions, *F*_*ST*_ fixation index within samples, *F*_*CT*_ fixation index between regions, *F*_*SC*_ fixation index among samples within regions

*** *P* = 0.00000 ± 0.00000

Given their genetic homogeneity, the samples from the State of Amazonas were joined in a single group (MN + CS + AU) and then compared with the samples from TU and AP (Table 5). The genetic distances (K-2P) between MN + CS + AU and TU and between TU and *A. nuneztovaris.s.* were 2.17 and 2.16 %, respectively. Oddly, between AP and *A. nuneztovaris.s.* it was 1.62 %. Between the samples of this study and *A. oswaldoi* species A, the genetic distances varied from 6.50 to 7.10 % (three times higher). The genetic distances among the three lineages (Table 6) varied from 1.60 (I and II) to 2.32 % (I and III). Between the three lineages and *A. nuneztovaris.s.* they ranged from 1.60 to 2.27 %, and between the three lineages and *A. dunhami* they ranged from 1.85 to 2.83 %. Between *A. nuneztovaris.s*. and *A. dunhami* the genetic distance was 2.55 %.

**Table 5:** Values of mean genetic distances (K-2P) and their respective standard errors (mean ± SE) among the five *Anopheles nuneztovari s.l.* samples from the Brazilian Amazon region and *Anopheles nuneztovari* *s.s*., *Anopheles dunhami* and the outgroup

Samples	MN + CS + AU	Tucuruí	Abacate da Pedreira	*A. nuneztovari* *s.s.*	*A. dunhami*	*A. oswaldoi* species A*
MN + CS + AU	*1.14* *%*					
Tucuruí	2.17 % ± 0.0042	*0.80* *%*				
Abacate da Pedreira	1.54 % ± 0.0035	1.51 % ± 0.0036	*0.61* *%*			
*A. nuneztovari* s. s.	2.01 % ± 0.0048	2.16 % ± 0.0052	1.62 % ± 0.0045	*0.05* *%*		
*A. dunhami*	2.70 % ± 0.0052	2.61 % ± 0.0054	2.00 % ± 0.0045	2.61 % ± 0.0058	*1.05* *%*	
*A. oswaldoi* species A*	7.10 % ± 0.010	6.92 % ± 0.0099	6.63 % ± 0.0098	6.78 % ± 0.010	6.50 % ± 0.0094	*1.66* *%*

In the diagonal, in *italics*: mean values intra-samples

*MN* Manaus, *CS* Careiro Castanho, *AU* Autazes, *K-2P* Kimura-2 Parameters, *Mean* *±* *SE* mean (in percentage) and respective standard error

* Outgroup

**Table 6:** Values of mean genetic distances (K-2P) and their respective standard errors (mean ± SE) among three *Anopheles nuneztovari s.l.* lineages from the Brazilian Amazon region and *Anopheles nuneztovari* s.s., *Anopheles dunhami* and the outgroup

Lineages	Lineage I	Lineage II	Lineage III	*A. nuneztovari* *s.s.*	*A. dunhami*	*A. oswaldoi* species A*
Lineage I	*1.20* *%*					
Lineage II	1.60 % ± 0.0044	*0.20* *%*				
Lineage III	2.32 % ± 0.0056	1.74 % ± 0.0044	*0.24* *%*			
*A. nuneztovari* s. s.	2.00 % ± 0.0054	1.60 % ± 0.0046	2.27 % ± 0.0059	*0. 15* *%*		
*A. dunhami*	2.67 % ± 0.0061	1.85 % ± 0.0046	2.83 % ± 0.0061	2.55 % ± 0.0058	*1.30* *%*	
*A. oswaldoi* species A*	7.28 % ± 0.1012	6.70 % ± 0.0095	7.03 % ± 0.0098	7.01 % ± 0.0096	6.50 % ± 0.0094	*0.30* *%*

For definition of the lineages, see ML tree topology (Fig. 3)

In the diagonal, in *italics*: mean values intra-groups

*K-2P* Kimura-2 Parameters, *Mean* *±* *SE* mean (in percentage) and respective standard error

* Outgroup

The average numbers of nucleotide substitution per site (*D*_xy_) between lineages were also calculated: I versus II, I versus III and II versus III were 1.60 % ± 0.00212, 2.30 % ± 0.00454 and 1.71 % ± 0.00273, respectively. Lineage I was separated from lineages II and III by zero and five fixed mutations, and two and zero shared mutations, respectively, whereas lineages II and III were separated by seven fixed mutations and 1 shared mutation (Additional file 2). The estimated time of divergence among lineages was of about 0.34 (lineages I and II) to 0.50 (I and III) million years. Between II and III it was ~0.37 million years (Additional file 2). These estimates indicate that they all diversified in the Pleistocene.

Additional file 3 shows the fixed differences (highlighted in red) among the haplotypes (H16–H18, H21–H23) of TU. The fixed differences are defined as sites at which all of the sequences in one sample are different from all of the sequences in a second sample.

*Anopheles dunhami* was identified for the first time in AU, representing a new record (Table 1) (see [31]).

### Microsatellite loci analyses

A total of 160 individuals encompassing the five samples were genotyped for 12 microsatellite loci (Additional file 4), totaling 1920 genotypes. Most of the loci were polymorphic, with a total of 171 alleles, varying from two (*Anu14, Anu15, Anu16*) to 25 (*Anu6*), except the *Anu14* locus that was monomorphic in the samples from TU and AP. The *Anu6* locus was the most polymorphic one, varying from 12 (TU) to 25 (AU) alleles. The samples from MN, CS and AU had the largest allele numbers (9.333; 8.000; 9.083, respectively) and allele richness (9.075; 7.852; 8.887, respectively) compared with the samples of TU (7.083; 6.922, respectively) and AP (6.750; 6.568, respectively). Of the 12 loci analysed, *Anu4*, *Anu10*, *Anu14* and *Anu28* were in HWE for all samples, whereas the other loci were in disequilibrium in at least one sample. Nineteen (31.67 %) out of the 60 comparisons exhibited Hardy–Weinberg disequilibrium, most of them suggesting heterozygote deficits. *LD* analysis was carried out to infer whether these deviations were due to the Wahlund effect, inbreeding, selection, genetic drift, gene flow or null alleles. Forty-one (12.42 %) out of 330 exact tests showed significant results for *LD* (*P* = 0.00076), after Bonferroni correction. Tucuruí had the greatest number of pair-loci (18) with significant test results, followed by AU (9) and CS (8). The other two samples showed three pair-loci, each of them with significant test results. A total of 55 private alleles distributed among the five samples were observed (Additional file 5). Autazes had the highest number of private alleles (17), but at low frequencies. Tucuruí and AP showed less private alleles (10 and 5; respectively); however, they exhibited higher frequencies (allele 218 of the locus *Anu6*, allele 292 of the *Anu12* and allele 218 of the *Anu25*).

Table 7 shows the genetic differentiation among the samples. As observed for the barcode region analysis, the three samples from the State of Amazonas were genetically similar (*F*_ST_ = 0.011–0.030), although the comparisons with AU showed statistically significant differences (*P* = 0.005). Higher and significant values (*P* = 0.005) were observed between the samples from Amazonas and TU (*F*_ST_ = 0.110-0.133), between the samples from Amazonas and AP (*F*_ST_ = 0.076–0.103) and between TU and AP (*F*_ST_ = 0.094), with a lower level of gene flow (*N*m = 6.05–3.25). AMOVA analysis including all samples revealed highly significant structures among them (*F*_ST_ = 0.081; *P* = 0.00000 ± 0.0000) (Table 4). The other hierarchical level revealed no significant structure among groups (*F*_CT_ = 0.060; *P* = 0.10178 ± 0.00339). There was isolation-by-distance (IBD) among the five samples (Mantel test: *r* = 0.906; *P* = 0.026500), covering a range of ~100–1211 km, indicating that ~90 % of the genetic differentiation observed is explained by geographic distance.

**Table 7:** Genetic distances based on the *Nm* (above the diagonal) and *F*_ST_ (below the diagonal) values among five *Anopheles nuneztovari s.l.* samples from the Brazilian Amazon region, based on the 12 microsatelite loci

Samples	Manaus	Careiro Castanho	Autazes	Tucuruí	Abacate da Pedreira
Manaus	–	43.83	15.99	3.25	4.33
Careiro Castanho	0.011	–	26.34	4.06	4.74
Autazes	0.030*	0.019*	–	3.63	6.05
Tucuruí	0.133*	0.110*	0.121*	–	4.82
Abacate da Pedreira	0.103*	0.100*	0.076*	0.094*	–

*N*m Mean number of migrant individuals per generation

* *P* = 0.005, after Bonferroni correction

**Fig. 4:**
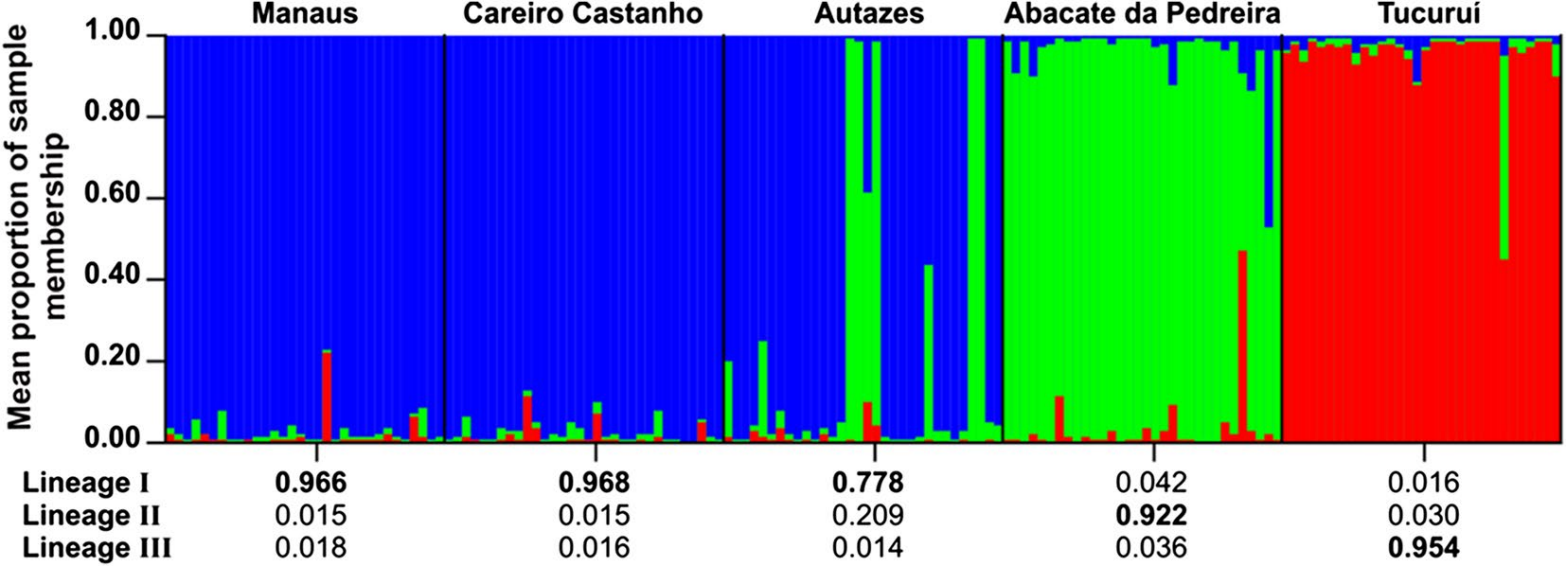
Bayesian genetic cluster analysis for the five *Anopheles nuneztovari* *s.l.* samples from the Brazilian Amazon region. Subdivision of all the individuals into K = 3 clusters. Group 1 (*blue*) comprises the three samples from the State of Amazonas; Group 2 (*green*) represents the sample of Abacate da Pedreira; Group 3 (*red*) represents the sample of Tucuruí

**Fig. 5:**
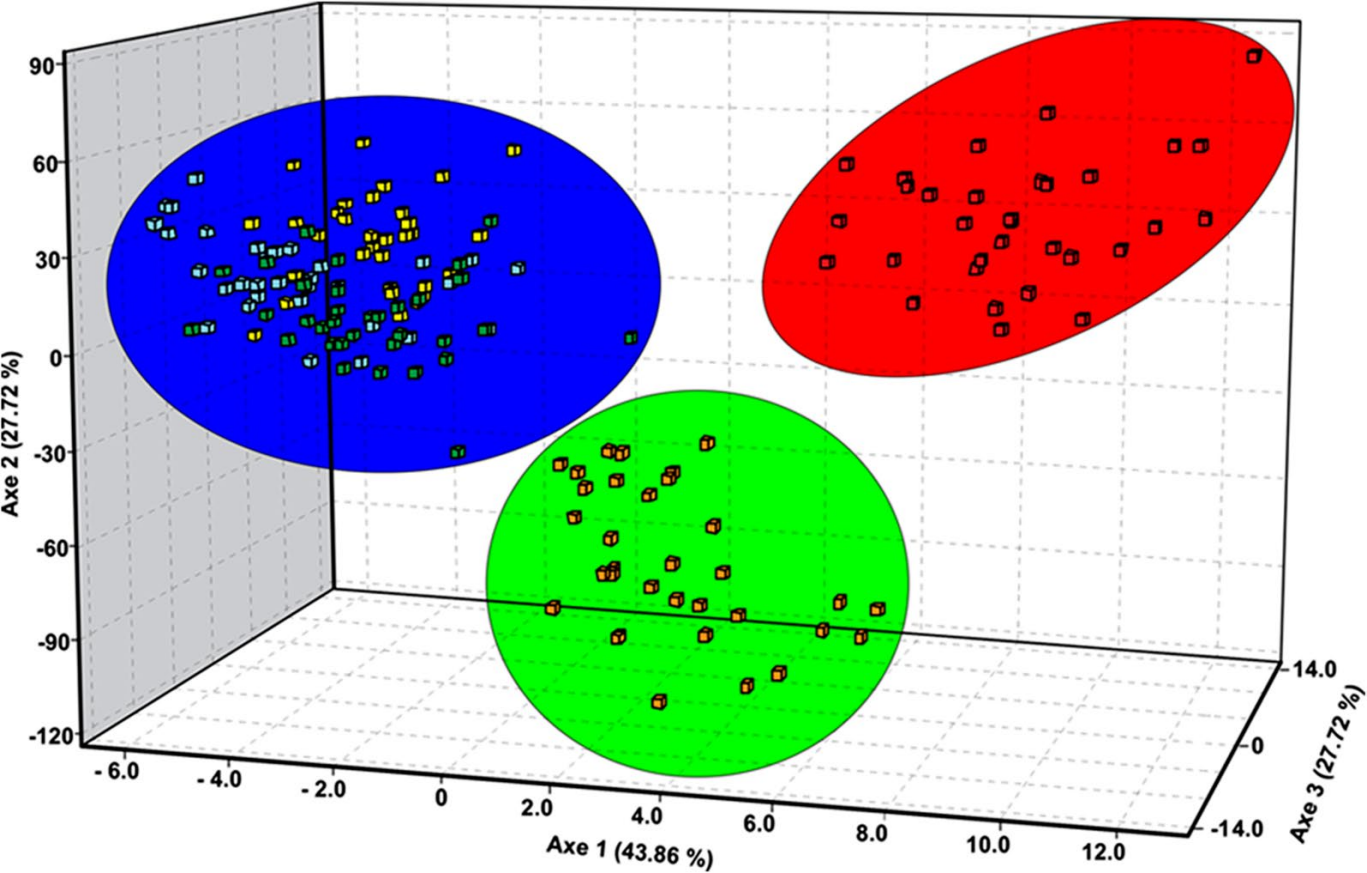
Factorial correspondence analysis of the five *Anopheles nuneztovari* *s.l.* samples from the Brazilian Amazon region, based on the 12 microsatellite loci. *Colors* represent the biological groups displayed in Fig. 4

Genetic structure analysis using a Bayesian approach (Fig. 4) identified three biological groups (*K* = 3), based on the highest mean value estimated log probability of data −6119.90 (SD = 7.20) and on the value close to the initial “plateau” of the curve, when *K* was plotted versus the mean posterior probability. These groups corresponded to the three samples from the State of Amazonas (blue), samples from AP (green) and from TU (red). However, five specimens from AU (blue) exhibited a larger proportion of their genomes (*q* > 0.980 average of five specimens) assigned to the green group instead of the blue group (Fig. 4). The samples from MN and CS (group 1) can be considered biologically pure (*Q* > 0.95 of belonging to the blue group) and so can those from TU (*Q* > 0.95 of belonging to the red group). Similarly, FCA analysis clearly separated three groups (Fig. 5), with the sample from TU being the most distant one. BAPS also identified three genetic groups (log ML = −6776.8164; posterior probability = 1.0) (Additional file 6).

## Discussion

Approximately half of the anopheline malaria vector species belong to sibling/cryptic species complexes [65]. In general, cryptic species [66] are of recent evolutionary origin and may, therefore, be morphologically very similar or identical, making their separation difficult. However, cryptic species can differ genetically, ecologically, behaviourally and epidemiologically. Over time, distinct morphological characters can also evolve; however, morphological differentiation tends to take longer, because changes in morphological traits require changes in multiple genes [67, 68]. In such a situation, integrated taxonomy with multiple markers is required for an accurate identification.

The two markers used in the present study were sensitive and concordant; both revealed three distinct genetic lineages for *A. nuneztovaris.l.* from the Brazilian Amazon region. These findings are strong enough to indicate that the populations of *A. nuneztovaris.l.* of this region do not belong to a single panmictic species, confirming previous evidence [17, 18, 21–24, 31].

In the phylogenetic analysis made using only sequences of this study, the ML tree retrieved five monophyletic groups, three of which (I, II, III) were represented by the five samples of this study. Group I was represented by the three samples from the central Amazon region and few specimens from AP. Group II comprised most of the specimens from the northeastern region (AP). Group III consisted of specimens from the eastern region (TU). These groups were confirmed by the Bayesian analyses (structure and BAPS) and FCA with microsatellite data.

The groups represented by lineages I and II (*A. goeldii*) were not well resolved (Fig. 3), likely due to the small number of informative sites between them for this marker (Additional file 3). Furthemore, there were shared haplotypes between AU and AP. The Bayesian analysis with microsatellite data (Fig. 4) showed very similar results; although this analysis clearly separated lineages I and II, five specimens from AU had a larger proportion of their genomes assigned to the AP sample. Taken together, these findings could reflect shared polymorphism or introgression caused by incomplete lineage sorting [65], a phenomenon often observed between young lineages and closely related species [69, 70], as may be the case of lineages I and II of this study.

Comparing the dataset of this study with the sequences from GenBank, the ML tree retrieved six groups, four of which (I, III, IV, V) were represented by specimens of the five localities of this study plus groups II (*A. nuneztovari* s.s.) and VI (*A. dunhami*) (Additional file 1). All sequences from GenBank identified as *A. goeldii* clustered with lineages I and II of this study, suggesting that they represent *A. goeldii*. In this case, *A. goeldii* was paraphyletic. If this is correct, the three groups could represent two or more species within *A. goeldii*. Intriguingly, these data suggest the groups I and III (*A. goeldii*) and II (*A. nuneztovaris.s*.) may be same species or incipient species. Supporting these findings, they also presented low genetic distances (1.60–2.0 %). A similar topology (BI tree) was obtained by Scarpassa and Conn [31], who reported that both samples from Bolivia/Colombia/Venezuela and the central region of the Brazilian Amazon clustered to the same major clade II. In contrast, previous studies [22, 24, 25] suggested that they are distinct species. Thus, these results could be explained by incomplete lineage sorting caused by retention of ancestral polymorphism due to the extremely recent divergence of this complex.

On the other hand, lineage III had high support in both ML analyses. Interestingly, no sequence from GenBank identified as *A. goeldii* clustered to lineage III (group IV), suggesting that it does not belong to *A. goeldii.* Furthermore, although all haplotypes were connected in the haplotype network (Fig. 2), there were no shared haplotypes between this lineage and lineage I, which could indicate some barrier to gene flow. Lineage III can be separated from lineages I and II by five to seven fixed differences, respectively. In the Bayesian analysis (Fig. 4), this lineage was considered biologically pure (*Q* > 0.95 of belonging to the red group), and in the FCA analysis (Fig. 5) it was the most distant one. Taken together, the lineage III may represent a new species within the Nuneztovari complex and may be following an evolutionary trajectory independent from the other lineages.

In the present study, the barcode region data indicated very high genetic structure and absence of gene flow (*N*m < 1) between the samples of the three regions. In contrast, genetic homogeneity was observed among the three samples from the State of Amazonas (lineage I). Therefore, specimens of these locations may belong to a unique species that is undergoing a differentiation process from lineage II, and distinct from lineage III.

The genetic distances (K-2P) among the three lineages were small (1.60–2.32 %) and very similar to those reported by Calado et al. [25], who also used the barcode region. However, the distance observed between lineages I and III was similar to those between *A. albitarsiss.s.* and *A. oryzalimnetes* (2.64 %) of the *A. albitarsis* complex [36] and between *A. dunhami* and *A. nuneztovaris.s.* (2.55–2.61 %) in this study. Ruiz-Lopes et al. [36] observed a threshold of 2.0 % for separating *A. albitarsis* H from *A. marajoara*, two sister taxa of the *A. albitarsis* complex, whereas the genetic distance between *A. triannulatuss.s*. and *A. halophylus*/*A. triannulatus* C varied from 1.7 to 2.30 % [69]. As observed, values >2.0 % have consistently been reported between sister taxa of species complexes in the *Nyssorhynchus* subgenus [31, 36, 37, 69–71], whereas the intra-specific divergence is rarely >2 %. These results suggest that the members of *A. nuneztovari* complex are of recent evolutionary origin, confirming previous studies [18, 31]. An example of recent divergence was reported in another vector insect, *Lutzomyia umbratilis*, that likely consists of two cryptic species which showed genetic distances from 0.8 to 1.4 % and moderately supported clades [72], suggesting recent diversification.

Most of the lineages observed in the present study were undetected in the isozymes study [22], except lineage III, likely because of their very recent divergence and the slow evolution rate of this marker for detecting incipient or recently diverged species. However, the sample of TU was the most divergent [22], indicating that its diversification may have started earlier. The results of this study are partially consistent with those obtained by ITS2 [17] and mtDNA-RFLP [18] that identified two groups in the Brazilian Amazon region. This partial disagreement is mainly attributable to differences in sampling strategies between these studies. Our findings, however, match those obtained with the *white* gene [24]. Lineages I and II of this study correspond to lineage 1 of Mirabello and Conn [24], whereas lineage III corresponds to lineage 4 [24]. This lineage is represented by samples from Altamira (State of Pará) and Areia Branca (State of Rondônia). Altamira is situated near to TU. The authors [24] reported two sympatric lineages, with no heterozygotes observed in either Altamira or Areia Branca. Similarly, the samples from TU and Areia Branca (Rondônia) shared haplotypes and clustered together, both in the haplotype network and in the BI tree analyses [31]. In the present study, an identical situation was observed: most of the specimens from TU clustered in lineage III, whereas the three others clustered in lineage II. Two of these three specimens shared haplotype (H19) with AP, suggesting that two sympatric species might exist in TU: the *A. goeldii* group (lineage II) and a new species (lineage III). The occurrence of two distinct genetic pools in TU could explain the highest number of pair-loci (18) in *LD* for the microsatellites data, which remained significant after the Bonferroni correction.

The diversification time estimated among the lineages falls in the Pleistocene Epoch (ranging from 0.34 to 0.50 myr), as previously observed [31], implying divergence within the last one million years. The most plausible hypothesis to explain the diversification among the lineages appears to be climatic changes, such as temperature fluctuations, reduced atmospheric CO_2_ and precipitation, occurred during the Pleistocene and which may have influenced the isolation of these populations in refuge areas, causing the differentiation between them by allopatry. Therefore, the two lineages or species sympatric observed in TU could represent secondary contact zones.

However, previous reports have suggested the Amazon river to be a significant barrier to dispersal for several species [73–75], including anophelines [76, 77]. The sampling of this study was not designed to test the predictions of the riverine barrier hypothesis, but some association may be possible. In this study, the largest differentiation was observed between lineages I and III. MN (lineage I) is situated on the north bank of the Amazon river, whereas CS, AU (both included in lineage I) and TU (lineage III) are located on the south bank. Curiously, mitochondrial and microsatellites markers revealed extensive gene flow, historical and contemporary, between MN and CS/AU, situated in opposite banks. One explanation for this finding is that in these localities the width of the river is not enough to prevent gene flow between populations. Alternatively, the dispersal of these mosquitoes may occur via passive transport, because around these localities, including the MN area, there is intense river traffic. In contrast, there was no gene flow between these locations and TU. Two interfluves (Xingu and Tapajós rivers) separate AU from TU, whereas three interfluves (Xingu, Tapajós and Madeira rivers) separate CS from TU, and these three and the Amazon river separate MN from TU. Therefore, it is possible that these interfluves may be acting as dispersal barriers for these anophelines.

Appreciable and significant mitochondrial and microsatellite differentiation was also observed between AP and TU, situated on opposite sides of the Amazon river delta (mouth). In this region, the Amazon river is widest and can reach up to 50 km in the rainy season. The Amazon river together with the Xingu, Araguaia and Tocantins and other smaller rivers form a large network in this region. This network may act as a dispersal barrier, even porous, restricting the contact between anophelines from the north and south banks, promoting genetic differentiation. Previous studies have reported differentiation between populations of *A. darlingi* [76, 77] and *A. marajoara* [71] in this region. On the other hand, the Mantel test showed that ~90 % of the genetic differentiation found among the five localities is explained by IBD, as observed for the *white* gene [24]. The localities sampled in this study may have influenced these results.

Taken together, the data clearly show that the three genetic lineages studied may be evolving independently in the Brazilian Amazon region. Lineages I and II may represent genetically distinct groups or species within *A. goeldii*, whereas lineage III may represent a new species and could be the most ancestral one in the Brazilian Amazon region.

Higher levels of intra-population genetic variability, estimated by the microsatellite loci, were detected for the central region (samples from the State of Amazonas) as compared to the samples from TU and AP, supporting previous findings of Scarpassa and Conn [31]. Based on these results, the authors proposed that the central Amazon region is likely to be the ancestral area of this species complex. In the present study, however, the higher level of genetic variability observed may be a consequence of an extensive contemporary gene flow among specimens of the localities of MN, CS and AU, which represent a panmictic population.

## Conclusions

The two markers used in this study were concordant; both revealed three distinct genetic lineages for *A. nuneztovaris.l.* in the Brazilian Amazon region, confirming previous reports. Lineages I and II may consist of genetically distinct groups (likely species) within *A. goeldii*. Lineage III could represent a new species of the *A. nuneztovari* complex and may be the most ancestral one in the Brazilian Amazon region. Since *A. nuneztovaris.l*. has been incriminated as a major local malaria vector in the State of Amapá [12], the lineage II (*A. goeldii*) is probably a malaria vector in that area. Furthermore, based on the previous reports [7, 9, 13] the lineage I (*A. goeldii*) likely may be involved in the malaria transmission in the central region from the Brazilian Amazon. The involvement of lineage III in the malaria transmission remains to be clarified. This study highlights the importance of utilizing integrative approaches for separate lineages and species of this complex, in order to achieve accurate results.

## Additional files

10.1186/s12936-016-1217-6 Maximum Likelihood (ML) topology tree of the 27 *Anopheles nuneztovari* *s.l.* haplotypes from the Brazilian Amazon region, two *Anopheles nuneztovari* *s.s.* haplotypes (H28, H29) and three *Anopheles dunhami* haplotypes (H31, H32, H33) and sequences downloaded from GenBank, using the GTR + I+G nucleotide substitution model. Values above each branch represent bootstrap support. *Anopheles oswaldoi* species A was used as outgroup.
10.1186/s12936-016-1217-6 Average numbers of nucleotide substitution per site (*D*_xy_), mean number of nucleotide differences (*K*), and the estimated time of divergence among the *Anopheles nuneztovari s.l.* lineages from the Brazilian Amazon region. * Sequence divergence estimates are given in percentage; divergence time estimates are given in million years, using mutation rates of 2.3 %.
10.1186/s12936-016-1217-6 Variable sites among 27 Barcode region haplotypes observed for *Anopheles nuneztovari* *s.l.* from the Brazilian Amazon region. H, Haplotypes.
10.1186/s12936-016-1217-6 Intra-population genetic diversity measures calculated for the five *Anopheles nuneztovari s.l.* samples from the Brazilian Amazon region, based on the 12 microsatellite loci. *N*_A_, Allele number; *A*_R_, Allele richness; *H*_O_, observed heterozygosity; *H*_E_, expected heterozygosity; *r*, estimated frequency of null alleles; *F*_IS_, inbreeding coefficient; –, no significant heterozygote deficiency; mono, monomorphic. The values in **bold** indicate Hardy–Weinberg disequilibrium at the loci, after Bonferroni correction (*P* = 0.004).
10.1186/s12936-016-1217-6 Private alleles observed in the five samples of *Anopheles nuneztovari* *s.l.* from the Brazilian Amazon region.
10.1186/s12936-016-1217-6 Bayesian analysis of population structure (BAPS) of the five *Anopheles nuneztovari* s.l. samples from the Brazilian Amazon region, using 12 microsatellite loci. Dataset analysis obtained from 160 specimens indicated the existence of three genetic clusters. Subdivision of all the specimens into K = 3 clusters. Cluster 1 (blue) comprises the specimens of three samples from the State of Amazonas; Cluster 2 (green) represents the sample from Abacate da Pedreira; Cluster 3 (red) represents the sample from Tucuruí.
