# Supplementary figures and images for: Evidence of new species for malaria vector Anopheles nuneztovari sensu lato in the Brazilian Amazon region
Source: Malar J. 2016 Apr 12;15:205. doi: 10.1186/s12936-016-1217-6 (PMC4828892; doi:10.1186/s12936-016-1217-6)

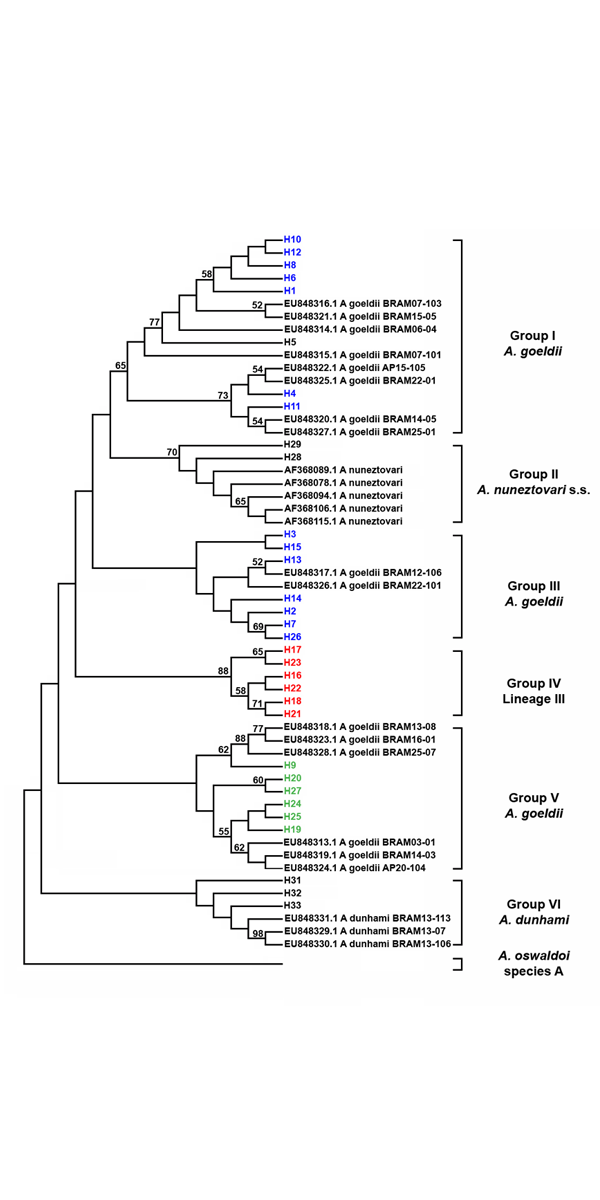

Supplement: Supplementary file 1 — 10.1186/s12936-016-1217-6 Maximum Likelihood (ML) topology tree of the 27 Anopheles nuneztovari s.l. haplotypes from the Brazilian Amazon region, two Anopheles nuneztovari s.s. haplotypes (H28, H29) and three Anopheles dunhami haplotypes (H31, H32, H33) and sequences downloaded from GenBank, using the GTR + I+G nucleotide substitution model. Values above each branch represent bootstrap support. Anopheles oswaldoi species A was used as outgroup. [file 12936_2016_1217_MOESM1_ESM.tif]

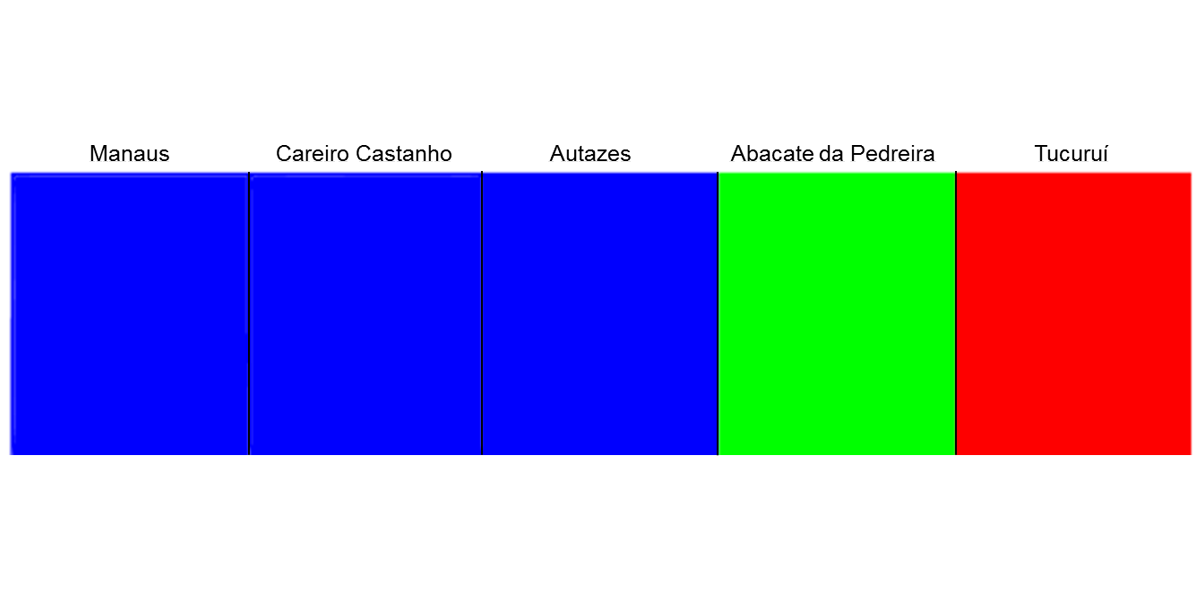

Supplement: Supplementary file 6 — 10.1186/s12936-016-1217-6 Bayesian analysis of population structure (BAPS) of the five Anopheles nuneztovari s.l. samples from the Brazilian Amazon region, using 12 microsatellite loci. Dataset analysis obtained from 160 specimens indicated the existence of three genetic clusters. Subdivision of all the specimens into K = 3 clusters. Cluster 1 (blue) comprises the specimens of three samples from the State of Amazonas; Cluster 2 (green) represents the sample from Abacate da Pedreira; Cluster 3 (red) represents the sample from Tucuruí. [file 12936_2016_1217_MOESM6_ESM.tif]
