# Supplementary material for: Evidence of new species for malaria vector Anopheles nuneztovari sensu lato in the Brazilian Amazon region
Source: Malar J. 2016 Apr 12;15:205. doi: 10.1186/s12936-016-1217-6 (PMC4828892; doi:10.1186/s12936-016-1217-6)
Supplement: Supplementary file 2 — 10.1186/s12936-016-1217-6 Average numbers of nucleotide substitution per site (D xy), mean number of nucleotide differences (K), and the estimated time of divergence among the Anopheles nuneztovari s.l. lineages from the Brazilian Amazon region. * Sequence divergence estimates are given in percentage; divergence time estimates are given in million years, using mutation rates of 2.3 %. [file 12936_2016_1217_MOESM2_ESM.doc]

**Additional file 2 Average numbers of nucleotide substitution per site (*D*_xy_), mean number of nucleotide differences (*K*) and the estimated time of divergence among the lineages of *Anopheles nuneztovari s.l.* from the Brazilian Amazon region**

| Lineages | Sequence*  divergence | Mean nucleotide differences (*K*) | Divergence  time | Number of fixed differences | Shared mutations |
| --- | --- | --- | --- | --- | --- |
| I *vs.* II | 1.60% | 14.44 | 0.34 | 0 | 2 |
| I *vs.* III | 2.30% | 15.54 | 0.50 | 5 | 0 |
| II *vs.* III | 1.71% | 11.31 | 0.37 | 7 | 1 |

* Sequence divergence estimates are given in percentage. Divergence time estimates are given in million years, using mutation rates of 2.3%.
