# Supplementary material for: Evidence of new species for malaria vector Anopheles nuneztovari sensu lato in the Brazilian Amazon region
Source: Malar J. 2016 Apr 12;15:205. doi: 10.1186/s12936-016-1217-6 (PMC4828892; doi:10.1186/s12936-016-1217-6)
Supplement: Supplementary file 3 — 10.1186/s12936-016-1217-6 Variable sites among 27 Barcode region haplotypes observed for Anopheles nuneztovari s.l. from the Brazilian Amazon region. H, Haplotypes. [file 12936_2016_1217_MOESM3_ESM.docx]

**Additional file 3 Variable sites observed among 27 haplotypes of the five samples analyzed of *Anopheles nuneztovari s.l.* from the Brazilian Amazon region**

| **H** |  |  |  |  |  |  |  |  | **1** | **1** | **1** | **1** | **1** | **1** | **2** | **2** | **2** | **2** | **2** | **3** | **3** | **3** | **3** | **3** | **3** | **4** | **4** | **4** | **4** | **4** | **4** | **4** | **5** | **5** | **6** | **6** | **6** | **6** | **6** | **6** |
| --- | --- | --- | --- | --- | --- | --- | --- | --- | --- | --- | --- | --- | --- | --- | --- | --- | --- | --- | --- | --- | --- | --- | --- | --- | --- | --- | --- | --- | --- | --- | --- | --- | --- | --- | --- | --- | --- | --- | --- | --- |
|  |  | **1** | **2** | **2** | **4** | **4** | **5** | **8** | **0** | **0** | **3** | **8** | **8** | **9** | **0** | **1** | **2** | **3** | **7** | **0** | **1** | **2** | **3** | **4** | **7** | **1** | **3** | **4** | **7** | **9** | **9** | **9** | **1** | **4** | **1** | **3** | **3** | **3** | **4** | **6** |
|  |  | **2** | **4** | **7** | **5** | **8** | **2** | **1** | **1** | **5** | **8** | **3** | **6** | **2** | **4** | **9** | **2** | **7** | **3** | **9** | **8** | **1** | **0** | **5** | **5** | **7** | **5** | **1** | **1** | **2** | **5** | **8** | **0** | **4** | **5** | **0** | **3** | **6** | **8** | **0** |
| **H1** |  | **T** | **A** | **C** | **G** | **T** | **T** | **T** | **G** | **T** | **T** | **A** | **G** | **G** | **A** | **G** | **T** | **C** | **C** | **A** | **A** | **A** | **G** | **C** | **G** | **G** | **A** | **A** | **G** | **C** | **A** | **A** | **A** | **T** | **C** | **A** | **A** | **C** | **T** | **C** |
| **H2** |  | **.** | **G** | **.** | **A** | **.** | **.** | **.** | **.** | **.** | **.** | **.** | **A** | **.** | **.** | **A** | **.** | **.** | **T** | **.** | **.** | **T** | **A** | **T** | **.** | **A** | **.** | **.** | **A** | **.** | **.** | **.** | **.** | **.** | **.** | **G** | **.** | **.** | **.** | **.** |
| **H3** |  | **.** | **G** | **.** | **A** | **A** | **.** | **.** | **.** | **.** | **.** | **.** | **A** | **.** | **.** | **A** | **.** | **.** | **T** | **.** | **.** | **T** | **A** | **T** | **.** | **.** | **.** | **.** | **A** | **.** | **.** | **.** | **G** | **.** | **T** | **G** | **.** | **.** | **.** | **.** |
| **H4** |  | **.** | **.** | **.** | **A** | **.** | **.** | **.** | **.** | **.** | **.** | **.** | **A** | **.** | **.** | **A** | **.** | **.** | **T** | **.** | **.** | **.** | **.** | **.** | **A** | **.** | **G** | **.** | **A** | **.** | **.** | **.** | **.** | **.** | **.** | **.** | **.** | **.** | **C** | **.** |
| **H5** |  | **.** | **.** | **.** | **.** | **.** | **.** | **.** | **.** | **.** | **.** | **.** | **.** | **.** | **.** | **.** | **.** | **T** | **T** | **.** | **.** | **.** | **.** | **.** | **.** | **.** | **.** | **.** | **.** | **T** | **.** | **.** | **.** | **.** | **.** | **.** | **.** | **.** | **.** | **.** |
| **H6** |  | **.** | **.** | **T** | **.** | **.** | **.** | **.** | **.** | **.** | **.** | **.** | **.** | **.** | **.** | **.** | **.** | **.** | **.** | **.** | **.** | **.** | **.** | **.** | **.** | **.** | **.** | **.** | **.** | **.** | **.** | **.** | **.** | **.** | **.** | **.** | **.** | **.** | **.** | **.** |
| **H7** |  | **.** | **G** | **.** | **A** | **.** | **.** | **.** | **.** | **.** | **.** | **.** | **A** | **.** | **.** | **A** | **C** | **.** | **T** | **.** | **.** | **T** | **A** | **T** | **.** | **A** | **.** | **.** | **A** | **.** | **.** | **.** | **.** | **.** | **.** | **G** | **.** | **.** | **.** | **.** |
| **H8** |  | **.** | **.** | **.** | **.** | **.** | **.** | **.** | **A** | **.** | **.** | **.** | **.** | **.** | **.** | **.** | **.** | **.** | **.** | **.** | **.** | **.** | **.** | **.** | **.** | **.** | **.** | **.** | **.** | **.** | **.** | **.** | **.** | **.** | **.** | **.** | **.** | **.** | **.** | **.** |
| **H9** |  | **.** | **G** | **.** | **A** | **.** | **.** | **.** | **.** | **.** | **.** | **.** | **A** | **.** | **.** | **A** | **.** | **.** | **T** | **.** | **.** | **T** | **A** | **T** | **A** | **.** | **.** | **.** | **A** | **.** | **G** | **.** | **.** | **.** | **.** | **G** | **.** | **.** | **C** | **C** |
| **H10** |  | **.** | **.** | **C** | **.** | **.** | **.** | **.** | **.** | **.** | **.** | **.** | **.** | **.** | **.** | **.** | **.** | **.** | **.** | **G** | **.** | **.** | **.** | **.** | **.** | **.** | **.** | **G** | **.** | **.** | **.** | **.** | **.** | **.** | **.** | **.** | **.** | **.** | **.** | **C** |
| **H11** |  | **.** | **.** | **.** | **A** | **.** | **.** | **.** | **.** | **.** | **.** | **.** | **A** | **.** | **.** | **A** | **.** | **.** | **T** | **.** | **.** | **.** | **.** | **.** | **.** | **.** | **G** | **.** | **A** | **.** | **.** | **.** | **.** | **.** | **.** | **.** | **.** | **.** | **C** | **.** |
| **H12** |  | **.** | **.** | **.** | **.** | **.** | **.** | **.** | **.** | **C** | **.** | **.** | **.** | **.** | **.** | **.** | **.** | **.** | **.** | **.** | **.** | **.** | **.** | **.** | **.** | **.** | **.** | **.** | **.** | **.** | **.** | **.** | **.** | **.** | **.** | **.** | **.** | **.** | **.** | **.** |
| **H13** |  | **.** | **G** | **.** | **A** | **.** | **.** | **.** | **.** | **.** | **.** | **.** | **A** | **.** | **.** | **A** | **.** | **.** | **T** | **.** | **.** | **T** | **A** | **T** | **.** | **A** | **G** | **.** | **A** | **.** | **.** | **.** | **.** | **.** | **.** | **G** | **.** | **.** | **.** | **.** |
| **H14** |  | **.** | **G** | **.** | **A** | **.** | **.** | **.** | **.** | **.** | **.** | **.** | **A** | **.** | **.** | **A** | **.** | **.** | **T** | **.** | **G** | **T** | **A** | **T** | **.** | **A** | **.** | **.** | **A** | **.** | **.** | **.** | **.** | **.** | **.** | **G** | **.** | **.** | **.** | **.** |
| **H15** |  | **.** | **.** | **.** | **A** | **.** | **.** | **.** | **.** | **.** | **.** | **.** | **A** | **.** | **.** | **A** | **.** | **.** | **T** | **.** | **.** | **T** | **A** | **.** | **.** | **.** | **.** | **.** | **A** | **.** | **.** | **.** | **.** | **.** | **.** | **G** | **.** | **.** | **.** | **.** |
| **H16** |  | **C** | **.** | **.** | **A** | **.** | **.** | **C** | **.** | **.** | **C** | **.** | **A** | **.** | **G** | **A** | **.** | **.** | **T** | **.** | **.** | **T** | **A** | **T** | **A** | **.** | **.** | **.** | **A** | **T** | **.** | **G** | **.** | **C** | **.** | **G** | **.** | **.** | **.** | **.** |
| **H17** |  | **C** | **.** | **.** | **A** | **.** | **.** | **C** | **.** | **.** | **C** | **.** | **A** | **.** | **G** | **A** | **.** | **.** | **T** | **.** | **.** | **T** | **A** | **T** | **A** | **.** | **.** | **.** | **A** | **T** | **.** | **.** | **.** | **C** | **.** | **G** | **.** | **.** | **.** | **.** |
| **H18** |  | **C** | **.** | **.** | **A** | **.** | **.** | **C** | **.** | **.** | **C** | **.** | **A** | **.** | **G** | **A** | **.** | **.** | **T** | **.** | **.** | **T** | **A** | **T** | **A** | **.** | **.** | **.** | **A** | **T** | **.** | **G** | **.** | **C** | **.** | **G** | **.** | **T** | **.** | **.** |
| **H19** |  | **.** | **G** | **.** | **A** | **.** | **.** | **.** | **.** | **.** | **.** | **G** | **A** | **.** | **.** | **A** | **.** | **.** | **.** | **.** | **.** | **T** | **A** | **T** | **A** | **.** | **.** | **.** | **A** | **.** | **.** | **.** | **.** | **.** | **.** | **G** | **.** | **T** | **C** | **.** |
| **H20** |  | **.** | **G** | **.** | **A** | **.** | **.** | **.** | **.** | **.** | **.** | **G** | **A** | **.** | **.** | **A** | **.** | **.** | **.** | **.** | **.** | **T** | **A** | **T** | **A** | **.** | **.** | **.** | **A** | **.** | **.** | **.** | **.** | **.** | **.** | **G** | **.** | **.** | **C** | **.** |
| **H21** |  | **C** | **.** | **.** | **A** | **.** | **.** | **C** | **.** | **.** | **C** | **.** | **A** | **.** | **G** | **A** | **.** | **.** | **T** | **.** | **.** | **T** | **A** | **T** | **A** | **.** | **.** | **.** | **A** | **T** | **.** | **G** | **.** | **C** | **.** | **G** | **G** | **T** | **.** | **.** |
| **H22** |  | **C** | **.** | **.** | **A** | **.** | **.** | **C** | **.** | **.** | **C** | **.** | **A** | **.** | **G** | **A** | **.** | **.** | **T** | **.** | **.** | **T** | **A** | **T** | **A** | **.** | **.** | **.** | **A** | **T** | **.** | **G** | **.** | **C** | **.** | **G** | **.** | **.** | **.** | **T** |
| **H23** |  | **C** | **.** | **.** | **A** | **.** | **C** | **C** | **.** | **.** | **C** | **.** | **A** | **.** | **G** | **A** | **.** | **.** | **T** | **.** | **.** | **T** | **A** | **T** | **A** | **.** | **.** | **.** | **A** | **T** | **.** | **.** | **.** | **C** | **.** | **G** | **.** | **.** | **.** | **.** |
| **H24** |  | **.** | **.** | **.** | **A** | **.** | **.** | **.** | **.** | **.** | **.** | **G** | **A** | **.** | **.** | **A** | **.** | **.** | **.** | **.** | **.** | **T** | **A** | **T** | **A** | **.** | **.** | **.** | **A** | **.** | **.** | **.** | **.** | **.** | **.** | **G** | **.** | **T** | **C** | **.** |
| **H25** |  | **.** | **G** | **.** | **A** | **.** | **.** | **.** | **.** | **.** | **.** | **.** | **A** | **.** | **.** | **A** | **.** | **.** | **.** | **.** | **.** | **T** | **A** | **T** | **A** | **.** | **.** | **.** | **A** | **.** | **.** | **.** | **.** | **.** | **.** | **G** | **.** | **T** | **C** | **.** |
| **H26** |  | **.** | **G** | **.** | **A** | **.** | **.** | **.** | **.** | **.** | **.** | **.** | **.** | **A** | **.** | **A** | **C** | **.** | **T** | **.** | **.** | **T** | **A** | **T** | **.** | **A** | **.** | **.** | **A** | **.** | **.** | **.** | **.** | **.** | **.** | **G** | **.** | **.** | **.** | **.** |
| **H27** |  | **T** | **.** | **.** | **A** | **.** | **.** | **.** | **.** | **.** | **.** | **G** | **A** | **.** | **.** | **A** | **.** | **.** | **.** | **.** | **.** | **T** | **A** | **T** | **A** | **.** | **.** | **.** | **A** | **.** | **.** | **.** | **.** | **.** | **.** | **G** | **.** | **.** | **C** | **.** |

H, haplotypes.
