# Supplementary material for: Evidence of new species for malaria vector Anopheles nuneztovari sensu lato in the Brazilian Amazon region
Source: Malar J. 2016 Apr 12;15:205. doi: 10.1186/s12936-016-1217-6 (PMC4828892; doi:10.1186/s12936-016-1217-6)
Supplement: Supplementary file 4 — 10.1186/s12936-016-1217-6 Intra-population genetic diversity measures calculated for the five Anopheles nuneztovari s.l. samples from the Brazilian Amazon region, based on the 12 microsatellite loci. N A, Allele number; A R, Allele richness; H O, observed heterozygosity; H E, expected heterozygosity; r, estimated frequency of null alleles; F IS, inbreeding coefficient; –, no significant heterozygote deficiency; mono, monomorphic. The values in bold indicate Hardy–Weinberg disequilibrium at the loci, after Bonferroni correction (P = 0.004). [file 12936_2016_1217_MOESM4_ESM.doc]

**Additional file 4 Intra-population genetic diversity measures calculated for the five samples of**

***Anopheles nuneztovari s.l.* from the Brazilian Amazon region based on the 12 microsatellites loci**

| **Locus** | Manaus  2N = 64 | Careiro Castanho  2N = 64 | Autazes  2N = 64 | Tucuruí  2N = 64 | Abacate da Pedreira  2N = 64 |
| --- | --- | --- | --- | --- | --- |
|  |  |  |  |  |  |
| Anu1 |  |  |  |  |  |
| *N*_A_ | 13 | 10 | 13 | 9 | 11 |
| *A*_R_ | 12.610 | 9.992 | 12.711 | 9.000 | 11.000 |
| *H*_O_ | 0.875 | 0.718 | 0.562 | 0.448 | 0.586 |
| *H*_E_ | 0.879 | 0.879 | 0.896 | 0.773 | 0.857 |
| P-HWE | 0.010 | 0.038 | **0.000** | **0.000** | **0.000** |
| r | – | – | 0.169 | 0.177 | 0.139 |
| F_IS_ | -0.011 | 0.170 | 0.362 | 0.410 | 0.304 |
|  |  |  |  |  |  |
| Anu4 |  |  |  |  |  |
| *N*_A_ | 13 | 11 | 13 | 8 | 4 |
| *A*_R_ | 12.595 | 10.516 | 12.522 | 7.871 | 4.000 |
| *H*_O_ | 0.875 | 0.750 | 0.562 | 0.967 | 0.687 |
| *H*_E_ | 0.688 | 0.613 | 0.657 | 0.795 | 0.565 |
| *P-HWE* | 0.975 | 0.996 | 0.029 | 0.295 | 0.249 |
| *r* | – | – | – | – | – |
| *F*_IS_ | -0.292 | -0.242 | 0.131 | -0.237 | -0.235 |
|  |  |  |  |  |  |
| Anu6 |  |  |  |  |  |
| *N*_A_ | 22 | 18 | 25 | 12 | 17 |
| *A*_R_ | 21.297 | 17.708 | 24.202 | 11.710 | 16.515 |
| *H*_O_ | 0.875 | 0.906 | 0.937 | 0.437 | 0.687 |
| *H*_E_ | 0.939 | 0.939 | 0.960 | 0.867 | 0.922 |
| *P-HWE* | 0.171 | 0.030 | **0.002** | **0.000** | **0.000** |
| *r* | – | – | 0.004 | 0.224 | 0.115 |
| *F*_IS_ | 0.054 | 0.020 | 0.008 | 0.488 | 0.243 |
|  |  |  |  |  |  |
| Anu9 |  |  |  |  |  |
| *N*_A_ | 3 | 3 | 3 | 5 | 5 |
| *A*_R_ | 3.000 | 3.000 | 2.993 | 4.932 | 4.899 |
| *H*_O_ | 0.093 | 0.187 | 0.218 | 0.645 | 0.406 |
| *H*_E_ | 0.518 | 0.569 | 0.466 | 0.622 | 0.399 |
| *P-HWE* | **0.000** | **0.000** | **0.000** | 0.270 | 0.280 |
| *r* | 0.276 | 0.239 | 0.165 | – | – |
| *F*_IS_ | 0.816 | 0.666 | 0.524 | -0.054 | -0.039 |
|  |  |  |  |  |  |
| Anu10 |  |  |  |  |  |
| *N*_A_ | 11 | 14 | 11 | 9 | 7 |
| *A*_R_ | 10.430 | 13.415 | 10.858 | 8.803 | 6.899 |
| *H*_O_ | 0.687 | 0.687 | 0.806 | 0.870 | 0.875 |
| *H*_E_ | 0.697 | 0.816 | 0.833 | 0.783 | 0.752 |
| *P-HWE* | 0.519 | 0.104 | 0.093 | 0.210 | 0.056 |
| *r* | – | – | – | – | – |
| *F*_IS_ | -0.001 | 0.145 | 0.016 | -0.130 | -0.181 |
|  |  |  |  |  |  |
| *Anu12* |  |  |  |  |  |
| *N*_A_ | 15 | 12 | 9 | 10 | 9 |
| *A*_R_ | 14.688 | 11.704 | 8.898 | 9.898 | 8.992 |
| *H*_O_ | 0.781 | 0.843 | 0.562 | 0.750 | 0.625 |
| *H*_E_ | 0.882 | 0.878 | 0.831 | 0.872 | 0.850 |
| *P-HWE* | 0.035 | 0.037 | **0.000** | 0.016 | **0.000** |
| *r* | – | – | 0.140 | – | 0.115 |
| *F*_IS_ | 0.101 | 0.025 | 0.313 | 0.127 | 0.253 |
|  |  |  |  |  |  |
| Anu14 |  |  |  |  |  |
| *N*_A_ | 2 | 2 | 2 | 1 | 1 |
| *A*_R_ | 1.906 | 1.906 | 1.993 | mono | mono |
| *H*_O_ | 0.031 | 0.031 | 0.062 | mono | mono |
| *H*_E_ | 0.031 | 0.031 | 0.061 |  |  |
| *P-HWE* | 1.000 | 1.000 | 1.000 |  |  |
| *r* | – | – | – |  |  |
| *F*_IS_ | -0.016 | -0.016 | -0.032 |  |  |
|  |  |  |  |  |  |
| Anu15 |  |  |  |  |  |
| *N*_A_ | 2 | 3 | 4 | 3 | 3 |
| *A*_R_ | 2.000 | 2.993 | 3.906 | 3.000 | 3.000 |
| *H*_O_ | 0.125 | 0.343 | 0.343 | 0.875 | 0.468 |
| *H*_E_ | 0.347 | 0.498 | 0.575 | 0.642 | 0.622 |
| *P-HWE* | **0.001** | 0.107 | **0.000** | **0.000** | **0.000** |
| *r* | 0.161 | – | 0.142 | 0.176 | 0.089 |
| *F*_IS_ | 0.634 | 0.299 | 0.394 | -0.383 | 0.235 |
|  |  |  |  |  |  |
| Anu16 |  |  |  |  |  |
| *N*_A_ | 4 | 4 | 2 | 6 | 2 |
| *A*_R_ | 3.899 | 3.993 | 2.000 | 5.985 | 2.000 |
| *H*_O_ | 0.218 | 0.343 | 0.129 | 0.656 | 0.187 |
| *H*_E_ | 0.475 | 0.528 | 0.177 | 0.727 | 0.380 |
| *P-HWE* | **0.000** | 0.008 | 0.233 | 0.006 | 0.007 |
| *r* | 0.169 | – | – | – | – |
| *F*_IS_ | 0.533 | 0.339 | 0.262 | 0.084 | 0.500 |
|  |  |  |  |  |  |
| Anu22 |  |  |  |  |  |
| *N*_A_ | 11 | 7 | 9 | 8 | 7 |
| *A*_R_ | 10.783 | 6.999 | 8.790 | 7.868 | 6.812 |
| *H*_O_ | 0.687 | 0.781 | 0.468 | 0.580 | 0.281 |
| *H*_E_ | 0.854 | 0.830 | 0.729 | 0.652 | 0.692 |
| *P-HWE* | 0.117 | 0.219 | **0.003** | 0.114 | **0.000** |
| *r* | – | – | 0.145 | – | 0.238 |
| *F*_IS_ | 0.182 | 0.045 | 0.347 | 0.095 | 0.588 |
|  |  |  |  |  |  |
| Anu25 |  |  |  |  |  |
| *N*_A_ | 7 | 4 | 7 | 5 | 6 |
| *A*_R_ | 6.711 | 3.999 | 6.891 | 5.000 | 5.899 |
| *H*_O_ | 0.437 | 0.687 | 0.406 | 0.448 | 0.500 |
| *H*_E_ | 0.539 | 0.581 | 0.720 | 0.495 | 0.773 |
| *P-HWE* | 0.339 | 0.417 | **0.000** | 0.467 | 0.014 |
| *r* | – | – | 0.177 | – | – |
| *F*_IS_ | 0.176 | -0.200 | 0.427 | 0.079 | 0.343 |
|  |  |  |  |  |  |
| Anu28 |  |  |  |  |  |
| *N*_A_ | 9 | 8 | 11 | 9 | 9 |
| *A*_R_ | 8.978 | 7.999 | 10.876 | 9.000 | 8.804 |
| *H*_O_ | 0.718 | 0.812 | 0.843 | 0.866 | 0.812 |
| *H*_E_ | 0.843 | 0.868 | 0.833 | 0.880 | 0.725 |
| *P-HWE* | 0.057 | 0.023 | 0.167 | 0.038 | 0.972 |
| *r* | – | – | – | – | – |
| *F*_IS_ | 0.134 | 0.050 | -0.029 | -0.001 | -0.138 |
|  |  |  |  |  |  |
| **Mean** |  |  |  |  |  |
| *N*_A_ | 9.333 | 8.000 | 9.083 | 7.083 | 6.750 |
| *A*_R_ | 9.075 | 7.852 | 8.887 | 6.922 | 6.568 |
| *H*_O_ | 0.533 | 0.590 | 0.491 | 0.685 | 0.555 |
| *H*_E_  *F*_IS_ | 0.641  0.192 | 0.669  0.148 | 0.644  0.226 | 0.737  0.043 | 0.685  0.170 |
|  |  |  |  |  |  |

# *N*_A_, Allele number; *A*_R_, Allele richness; *H*_O_, observed heterozygosity; *H*_E_, expected heterozygosity; *r*, estimated frequency of null alleles; *F*_IS_, inbreeding coefficient; –, no significant heterozygote deficiency; mono, monomorphic. The values in bold indicate disequilibrium in the loci according to Hardy-Weinberg Equilibrium, after the Bonferroni correction (*P* = 0.004).
