# Supplementary material for: Evidence of new species for malaria vector Anopheles nuneztovari sensu lato in the Brazilian Amazon region
Source: Malar J. 2016 Apr 12;15:205. doi: 10.1186/s12936-016-1217-6 (PMC4828892; doi:10.1186/s12936-016-1217-6)
Supplement: Supplementary file 5 — 10.1186/s12936-016-1217-6 Private alleles observed in the five samples of Anopheles nuneztovari s.l. from the Brazilian Amazon region. [file 12936_2016_1217_MOESM5_ESM.doc]

Additional file 5 Private alleles observed in the five samples of *Anopheles nuneztovari* *s.l.* from the Brazilian Amazon region

| Locus | Manaus | Careiro Castanho | Autazes | Tucuruí | Abacate da Pedreira | Total |
| --- | --- | --- | --- | --- | --- | --- |
| *Anu1* | 289 (0.031) 291 (0.047) | 287 (0.063) | 245 (0.031) 293 (0.016) | 265 (0.017) | – | 6 |
| *Anu4* | 222 (0.016) 282 (0.016) 291 (0.016) | 285 (0.016) 294 (0.016) | 216 (0.016) 231 (0.016) | – | – | 7 |
| *Anu6* | – | 228 (0.063) | 200 (0.047) 202 (0.094) 210 (0.016) 250 (0.031) 252 (0.031) 254 (0.031) 256 (0.031) | 216 (0.078) 218 (0.172) 224 (0.016) 284 (0.031) | 240 (0.016) 288 (0.063) | 14 |
| *Anu9* | – | – | – | 287 (0.016) | 291 (0.016) | 2 |
| *Anu10* | 217 (0.016) 219 (0.016) | 231 (0.016) 243 (0.016) | 212 (0.032) | – | – | 5 |
| *Anu12* | 256 (0.016) 280 (0.063) 296 (0.031) | 252 (0.031) 264 (0.016) | 258 (0.016) | 292 (0.094) | – | 7 |
| *Anu14* | – | – | – | – | – | – |
| *Anu15* | – | 215 (0.031) | 225 (0.016) |  |  | 2 |
| *Anu16* | – | 291 (0.078) | – | 299 (0.031) 317 (0.047) | – | 3 |
| *Anu22* | 269 (0.016) | – | – | – | 299 (0.016) | 2  10 |
| *Anu25* | 233 (0.016) | – | 260 (0.031) | – | 218 (0.188) | 3 |
| *Anu28* | 141 (0.031) | – | 171 (0.016) 175 (0.031) | 163 (0.050) | – | 4 |
| Total | 13 | 10 | 17 | 10 | 5 | 55 |
|  |  |  |  |  |  |  |
